# Ischemic wound revascularization by the stromal vascular fraction relies on host-donor hybrid vessels

**DOI:** 10.1038/s41536-023-00283-6

**Published:** 2023-02-11

**Authors:** Roman Vuerich, Elena Groppa, Simone Vodret, Nadja Annelies Ruth Ring, Chiara Stocco, Fleur Bossi, Chiara Agostinis, Matteo Cauteruccio, Andrea Colliva, Mohammad Ramadan, Francesca Simoncello, Federica Benvenuti, Anna Agnelli, Franca Dore, Flavia Mazzarol, Massimo Moretti, Alice Paulitti, Silvia Palmisano, Nicolò De Manzini, Mattia Chiesa, Manuel Casaburo, Angela Raucci, Daniela Lorizio, Giulio Pompilio, Roberta Bulla, Giovanni Papa, Serena Zacchigna

**Affiliations:** 1 Cardiovascular Biology Laboratory, International Centre for Genetic Engineering and Biotechnology (ICGEB), Trieste, Italy; 2 Department of Life Sciences, University of Trieste, Trieste, Italy; 3 Department of Medical, Surgical and Health Sciences, University of Trieste, Trieste, Italy; 4 Plastic Reconstructive and Aesthetic Surgery Department, Ospedale di Cattinara, ASUGI, 34149 Trieste, Italy; 5 Institute for Maternal and Child Health, Istituto di Ricovero e Cura a Carattere Scientifico (I.R.C.C.S.) “Burlo Garofolo”, Trieste, Italy; 6 Cellular Immunology Laboratory, International Centre for Genetic Engineering and Biotechnology (ICGEB), Trieste, Italy; 7 Nuclear Medicine Unit, University Hospital of Trieste—ASUGI, Trieste, Italy; 8 VivaBioCell S.p.A., Udine, Italy; 9 Centro Cardiologico Monzino IRCCS, Milano, Italy; 10 Department of Biomedical, Surgical and Dental Sciences, University of Milano, 20122 Milano, Italy; 11 Present Address: Scuola Internazionale Studi Superiori Avanzati (SISSA), 34136 Trieste, Italy; 12 Present Address: Ludwig Boltzmann Research Group SHoW—Senescence and Healing of Wounds, LBI Trauma, Vienna, Austria

**Keywords:** Biological therapy, Regeneration

## Abstract

Nonhealing wounds place a significant burden on both quality of life of affected patients and health systems. Skin substitutes are applied to promote the closure of nonhealing wounds, although their efficacy is limited by inadequate vascularization. The stromal vascular fraction (SVF) from the adipose tissue is a promising therapy to overcome this limitation. Despite a few successful clinical trials, its incorporation in the clinical routine has been hampered by their inconsistent results. All these studies concluded by warranting pre-clinical work aimed at both characterizing the cell types composing the SVF and shedding light on their mechanism of action. Here, we established a model of nonhealing wound, in which we applied the SVF in combination with a clinical-grade skin substitute. We purified the SVF cells from transgenic animals to trace their fate after transplantation and observed that it gave rise to a mature vascular network composed of arteries, capillaries, veins, as well as lymphatics, structurally and functionally connected with the host circulation. Then we moved to a human-in-mouse model and confirmed that SVF-derived endothelial cells formed hybrid human-mouse vessels, that were stabilized by perivascular cells. Mechanistically, SVF-derived endothelial cells engrafted and expanded, directly contributing to the formation of new vessels, while a population of fibro-adipogenic progenitors stimulated the expansion of the host vasculature in a paracrine manner. These data have important clinical implications, as they provide a steppingstone toward the reproducible and effective adoption of the SVF as a standard care for nonhealing wounds.

## Introduction

Nonhealing wounds represent a major clinical burden, with a prevalence of 2–5%, similar to that of heart failure^1–4^. A chronic wound is defined as a full-thickness skin defect that fails to heal after 3 months of standard care. It generally arises from a vascular, diabetic or pressure ulcer, but can have less frequent aetiologies, including infections, immune diseases, traumas, burns and post-surgery complications. The main reason why an ulcer does not heal and become chronic is the existence of co-morbidities, such as diabetes or peripheral artery disease, which eventually result in inadequate vascularization and lack of trophic support^5^. Poor vascularization also accounts for the failure of both skin grafts and dermal substitutes, which is an integral limitation of these emerging therapeutic opportunities for wound treatment^6,7^.

Over the past decade, the use of the autologous stromal vascular fraction (SVF) from the adipose tissue has been proposed to overcome this limitation^8^. The SVF is a heterogeneous population including endothelial cells (ECs), mesenchymal progenitors, inflammatory cells, pericytes, and smooth muscle cells, which can be easily obtained from the adipose tissue^9^. The capacity of the SVF to promote the formation of new blood vessels has been documented by a few clinical trials, which have assessed the therapeutic potential of the SVF in patients affected by both peripheral artery disease and nonhealing wounds. These studies provided inconclusive results, largely because they included a small number of patients, lacked a placebo control group, and used different protocols to isolate, expand, dose and inject the cells^8,10–12^. Many of these studies ended by highlighting these limitations and warranting future work aimed at characterizing the SVF composition, describing its mechanism of action and establishing the optimal dose and administration route^9,13^. This appears to be an essential requirement to obtain reproducible results and allow the adoption of the SVF as a standard therapy in routine clinical practice.

To address these gaps, we set up a new model of the ischemic wound in mice, able to recreate the clinical condition. In this model, we investigated the mechanism of action of the SVF, using both a mouse-in-mouse approach, to genetically trace the transplanted cells and assess their contribution to new vessel formation, and a human-in-mouse approach to confirm the clinical relevance of our findings.

## Results

### SVF cells layered on a clinical-grade scaffold directly contribute to the formation of new vessels in a new model of ischemic wound

**Fig. 1:**
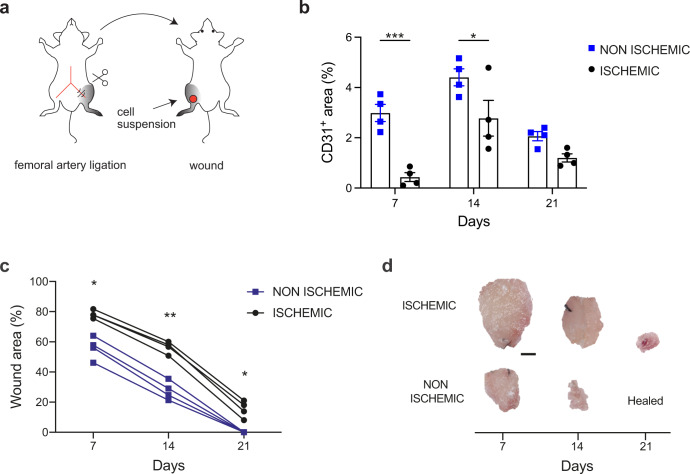
A new in vivo model of the ischemic wound. **a** Schematic representation of a new murine model of chronic ischemic wound. The left femoral artery is ligated to induce hindlimb ischemia. The animal is then turned upside down and a full-thickness skin punch wound is created above the ischemic muscle, on the same side. **b** Quantification of CD31^+^ area in ischemic and nonischemic wounds at the indicated time points. **c** Quantification of wound area in ischemic and nonischemic limbs, treated with an INTEGRA scaffold, at the indicated time points. **d** Representative images of wounds as quantified in panel **c**. Data are shown as individual values in (**b**, **c**); *n* = 4 per group. Scale bar in (**d**), 1 mm. Statistical significance was determined using two-way ANOVA for repeated measurements in (**b**, **c**). *P < 0.05, **P < 0.01, ***P < 0.001.

We set up a novel mouse model of the ischemic wound by excising the left femoral artery, followed by full-thickness wounding of the skin above the posterior femoralis biceps muscle, with the animal in a prone position (Fig. 1a). Limb ischemia resulted in a significant reduction in the number of CD31^+^ vessels (Fig. 1b), paralleled by a marked delay in wound closure (Fig. 1c). As shown in Fig. 1d, wounds at day 21 healed completely in normoperfused, but not in ischemic limbs.

In this model, we assessed the capacity of the mouse SVF to promote neo-vascularization and wound healing. To establish the best route of administration, we isolated the SVF from transgenic mice constitutively expressing membrane-localized tdTomato (mT). We either injected mT^+^ SVF cells in the subcutaneous tissue surrounding the wound perimeter, to mimic the procedure used by the majority of clinical trials, or seeded them on a clinical-grade scaffold prior to its application on the wound bed. Among the various skin substitutes, we chose INTEGRA, as it outperformed over a few alternative wound dressing devices available on the market and it is extensively used in the clinics^14^. As shown in Supplementary Fig. 1a, b, the engraftment of transplanted cells was significantly higher in the case of cell application on INTEGRA, which resulted massively colonized by mT^+^ cells at day 7. Therefore, we combined SVF cell transplantation with INTEGRA in all next experiments.

**Fig. 2:**
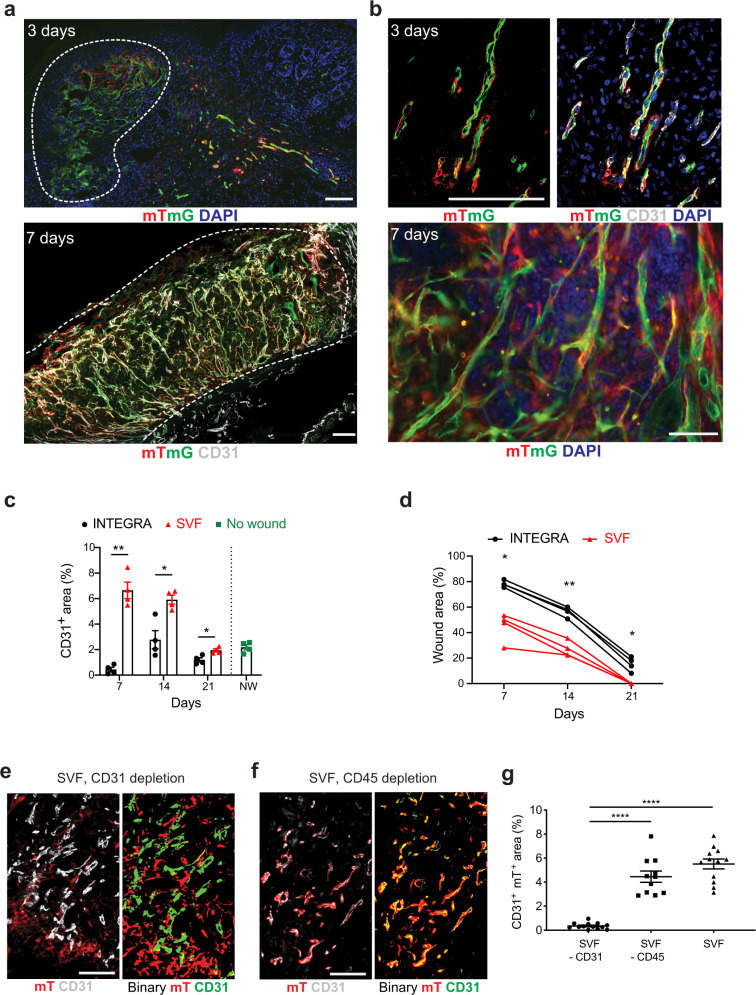
SVF promotes angiogenesis an in vivo model of the ischemic wound. **a** Representative images of INTEGRA scaffold populated by Cdh5-CreER;mTmG SVF cells at the indicated time points. In the upper panel, mTmG^+^ cells are located both within the scaffold, marked by a dashed white line, and in the muscle underneath. In the lower panel, CD31 staining identifies both host (CD31^+^ EGFP^−^) and donor (CD31^+^ EGFP^+^) vessels. **b** High-magnification images of the same tissues as in (**a**) to visualize mTmG^+^ CD31^+^ vessels and the increased complexity of SVF-formed vascular network over time. Upper panels are from outside, whereas lower panel is from within the INTEGRA scaffold. **c** Quantification of CD31^+^ area upon application of INTEGRA scaffold, either alone or in combination with SVF cells, at the indicated time points. Data are compared to those of a healthy skin. **d** Quantification of ischemic wound area upon application of INTEGRA scaffold, either alone or in combination with SVF cells, at the indicated time points. **e** Representative image of INTEGRA scaffold populated by Cdh5-CreER;mTmG SVF cells upon depletion of CD31^+^ ECs (left panel). The right panel shows the corresponding binary image, in which mT signal is in red and CD31 signal is in green. The minimal overlap between the two colors indicates almost null contribution of the transplanted cells to vessel formation. **f** Representative image of INTEGRA scaffold populated by Cdh5-CreER;mTmG SVF cells upon depletion of CD45^+^ inflammatory cells (left panel). The right panel shows the corresponding binary image, in which mT signal is in red and CD31 signal is in green. The large overlap between the two colors indicates the massive contribution of the transplanted cells to vessel formation. **g** Quantification of the area covered by CD31^+^ mT^+^ cells within INTEGRA at 7 days, normalized on the whole scaffold area. Data are shown as individual values in (**c**, **d**, **g**); *n* ≥ 3 per group. Scale bar in (**a**, **b**, **e**, **f**), 100 μm. Statistical significance was determined using two-way ANOVA for repeated measurements in (**c**, **d**), and one-way ANOVA followed by Tukey’s multiple comparison test in (**g**). *P < 0.05, **P < 0.01, ****P < 0.0001.

To elucidate whether ECs in the SVF were able to engraft and survive upon application on the ischemic wound, we used a genetic lineage tracing system to follow the fate of both ECs and the other SVF cells. We purified the SVF from Cdh5-CreER/mTmG mice, in which mT labels all cells, while the Cdh5, exclusively active in ECs, activates a switch to membrane-EGFP upon tamoxifen-inducible Cre recombination (Supplementary Fig. 2a). Donor SVF was isolated from these mice 7 days after tamoxifen administration, seeded on INTEGRA and implanted on the ischemic wound of recipient wild type mice. This model allowed us to track all transplanted cells, as they were fluorescently labeled, and, among these, to discriminate between ECs (green, mG^+^) and other cell types of the SVF (red, mT^+^). Histological analysis at day 3 revealed many mT^+^ cells and mG^+^ ECs that colonized the scaffold and migrated into the muscle underneath, forming elongated vascular structures (Fig. 2a, upper panel). These structures were further extended and organized in a complex vascular network at day 7 (Fig. 2a, lower panel and 3D model in Supplementary Movie 1), with almost 30 ± 9% proliferating ECs (Supplementary Fig. 3). Higher magnification images showed that at day 3 mT^+^ cells spontaneously wrapped around mG^+^ endothelial tubes (Fig. 2b). These mT^+^ cells persisted in the complex network observed at 7 days, consistent with the ability of the SVF to orchestrate the formation of mature vessels composed of both endothelial and perivascular cells (Fig. 2b and Supplementary Movie 2). To evaluate the therapeutic impact of the SVF, we monitored wound closure and vessel density at multiple time points, until wound closure. As shown in Fig. 2c, d, SVF-treated wounds showed a remarkable increase in the number of vessels at all time points, resulting in improved wound healing. While vessel density remained reduced in ischemic control wounds at 21 days, it was restored to a normal level in SVF-treated wounds (Fig. 2c for quantification and Supplementary Fig. 2b, c for representative images). The increased vascularization also promoted full re-epithelization similar to a healthy skin, suggesting regeneration of hair follicles (Supplementary Fig. 2c). Implantation of the SVF in normoxic wounds had minimal effect on wound closure rate and slightly increased vessel density (Supplementary Fig. 4).

Next, we characterized the composition of the mouse SVF, which was composed by hematopoietic, mesenchymal and ECs (Supplementary Fig. 5). By depleting ECs from the SVF using anti-CD31 magnetic beads prior to its implantation, we observed no vessels of donor origin (the percentage of CD31^+^ area co-localizing with mT signal was almost null (images in Fig. 2e and quantification in Fig. 2g). In contrast, the depletion of CD45^+^ inflammatory cells did not affect the capacity of the SVF to vascularize the scaffold, with a number of mT^+^ CD31^+^ transplanted cells comparable to the one obtained by the whole SVF (Fig. 2f, g).

Thus, SVF-derived ECs are required to form new vessels that persist up to 21 days and promote wound healing, while SVF-derived inflammatory cells are dispensable.

### The stromal vascular fraction contributes to the formation of full vascular units which integrate with host vessels

**Fig. 3:**
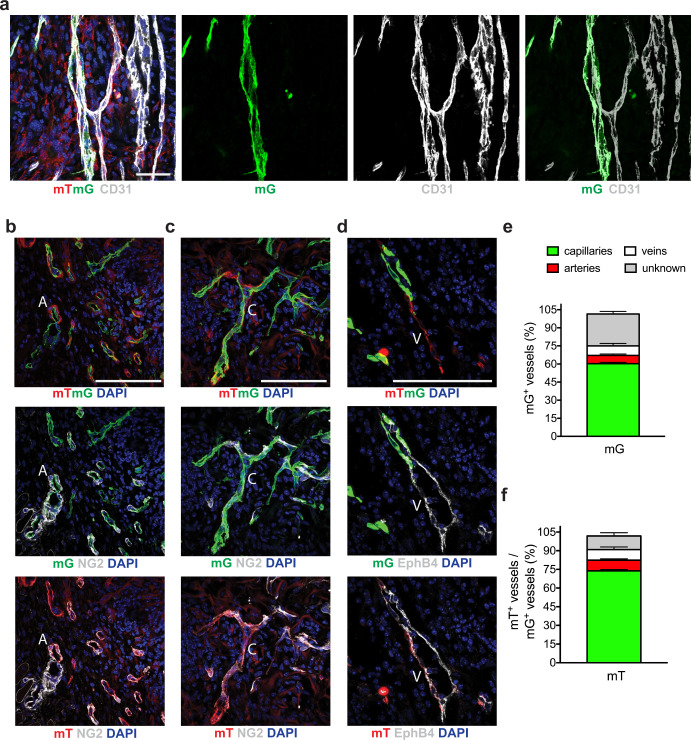
SVF cells promote the formation of a hierarchical vascular network integrated with host vessels. **a** Representative image of Cdh5-CreER;mTmG SVF-formed vessels at day 7 after cell implantation. CD31 staining labels both donor (CD31^+^ EGFP^+^) and host (CD31^+^ EGFP^−^) ECs. Separated channels on the right clearly indicate the formation of hybrid vessels, composed of both EGFP^+^ (donor) and EGFP^−^ (host) ECs. **b** Representative image of Cdh5-CreER;mTmG SVF-formed vessels at day 7 after cell implantation, stained for NG2 to visualize the formation of a large and mature artery (A), wrapped by a thick layer of NG2^+^ pericytes, which are partially positive for non-endothelial SVF-derived mT. **c** Representative image of Cdh5-CreER;mTmG SVF-formed vessels at day 7 after cell implantation, stained for NG2 to visualize the formation of a long and thin capillary (C), wrapped by a few sparse NG2^+^ pericytes. **d** Representative image of Cdh5-CreER;mTmG SVF-formed vessels at day 7 after cell implantation, stained for EphB4 to visualize the formation of a large vein (V). **e** Quantitative analysis of the different vessel types formed by SVF-derived mG^+^ EC at day 7 after implantation of SVF isolated from Cdh5-CreER;mTmG mice. **f** Quantitative analysis of the different vessel types that contain mT^+^ perivascular cells at day 7 after implantation of SVF cells derived from Cdh5-CreER;mTmG mice. Data in (**e**, **f**) are shown as mean ± SEM. *n* ≥ 3 per group. Scale bar in (**a**–**d**) is 100 μm.

We exploited our genetic tracing system to assess whether SVF-derived vessels can connect with the host vasculature at the site of implantation. We observed numerous hybrid vessels, which were composed by both mG^+^ (donor) and mG^−^ (host) CD31^+^ ECs, as shown in Fig. 3a. This indicated that donor and host ECs jointly formed the new vessels.

We then sought to understand whether the injected mG^+^ ECs contributed to the generation of the various vessel types that normally compose a vascular unit, namely arteries, capillaries, and veins. We used a panel of markers to discriminate among these vessel types. As shown in Supplementary Fig. 6a, α-SMA staining discriminates between positive large vessels (arteries and veins) and α-SMA^−^ capillaries. Veins can be distinguished using the venous endothelial marker EphB4, while arteries and capillaries stain positive for the perivascular marker NG2. In summary, veins are α-SMA^+^ and EphB4^+^, arteries are α-SMA^+^ and NG2^+^, and capillaries are α-SMA^−^ and NG2^+^.

Using this immunophenotyping strategy, we found that of the implanted SVF cells, 60% contributed to the formation of capillaries, 8% to veins and 7% to arteries. This is shown by representative images for each vessel type in Fig. 3b–d and quantification in Fig. 3e. This distribution mimics the physiological composition of a cutaneous vascular tree (Supplementary Fig. 6b, c).

Numerous cells wrapping the neo-vessels expressed mT, indicating their origin from the non-endothelial compartment of the SVF. These mT^+^ cells were consistently CD31^−^, yet could be detected in all major vessel types, often scoring positive for NG2 in capillaries and arteries and frequently in close contact with EphB4^+^ ECs in veins (Fig. 3b–d for representative images and Fig. 3f for quantification). Based on their localization and expression of NG2, we presume that most SVF-derived non-ECs that were incorporated in neo-vessels were in fact pericytes and smooth muscle cells. In accordance, when we implanted pure ECs in vivo, the neo-vessels appeared nude, lacking supportive perivascular coverage (Supplementary Fig. 7).

Thus, the SVF contributes to the formation of a complex vascular network, in which both ECs and perivascular cells of both donor and host origin interconnect to generate hybrid vascular structures. A high-magnification image, showing SVF-derived ECs connecting with a host EphB4^+^ vein is shown in Supplementary Fig. 8.

**Fig. 4:**
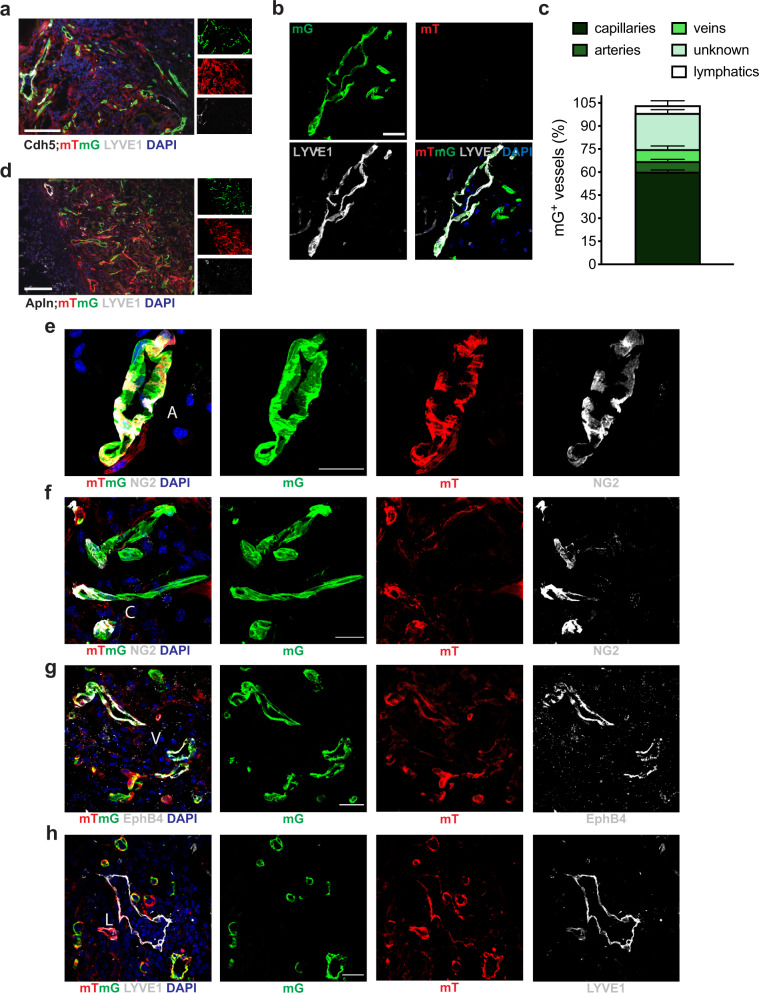
Blood and lymphatic vessels formed by the SVF have different cellular origins. **a** Representative image of Cdh5-CreER;mTmG SVF-formed vessels at day 7 after cell implantation, stained for LYVE-1 to visualize the formation of lymphatic vessels. **b** High magnification of Cdh5-CreER;mTmG SVF-formed vessels showing co-localization between LYVE-1 and mG signals. **c** Quantification of the contribution of mG^+^ ECs derived from Cdh5-CreER;mTmG SVF to the formation of the different vessel types, including lymphatics, at day 7 after cell implantation. Data are shown as mean ± SEM. *n* ≥ 3 per group. **d** Representative image of Apln-CreER;mTmG SVF-formed vessels at day 7 after cell implantation, stained for LYVE-1. **e** High magnification of a Apln-CreER;mTmG SVF-formed artery (A), showing mG^+^ ECs wrapped by a thick layer of NG2^+^ perivascular cells. **f** High magnification of Apln-CreER;mTmG SVF-formed capillaries (C), showing mG^+^ ECs wrapped by sparse and discontinuous NG2^+^ perivascular cells. **g** High magnification of Apln-CreER;mTmG SVF-formed veins (V), containing mG^+^ EphB4^+^ ECs. **h** High magnification of Apln-CreER;mTmG SVF-formed lymphatics (L), which are formed by mG^−^ LYVE-1^+^ mT^+^ ECs. Scale bar in (**a**, **d**) is 100 μm; in (**b**, **e**, **f**, **g**, **h**) is 25 μm.

We were intrigued by the fact that about 25% of the SVF-derived vessels did not exhibit a clear identity. Because these vessels were largely deprived of perivascular cells, we hypothesized that they could be lymphatics. Indeed, about 20% of these vessels scored positive for the lymphatic marker LYVE-1 (shown at two different magnifications in Fig. 4a, b and quantified in Fig. 4c). Because recent evidence indicates a certain degree of plasticity between blood and lymphatic ECs^15^, we used an alternative tracing system, in which tamoxifen-induced recombination happens selectively in pro-angiogenic ECs in blood vessels, which express Apelin (Apln)^16,17^. We harvested the SVF from Apln-CreER/mTmG mice and applied on wounded wild type animals, as described. In this case, tamoxifen was administered to recipient mice immediately after cell transplantation, to label Apln^+^ sprouting ECs. While Apln^+^ cells gave rise to arteries, capillaries and veins with a distribution similar to the one observed in Cdh5-CreER/mTmG mice, they were completely absent from LYVE-1^+^ vessels (shown at different magnifications in Fig. 4d–h). The fact that Apln^+^ ECs were confined to blood vessels indicates that blood and lymphatic structures formed by the SVF had an independent origin and that no transdifferentiation occurred between the two EC lineages.

### Human stromal vascular fraction promotes wound revascularization

To validate our data in a clinically relevant setting, we characterized the human SVF upon its ex vivo expansion, to obtain a high number of cells, suitable for human therapy. We harvested the SVF from subcutaneous lipoaspirate from seven healthy individuals (donor characteristics are reported in Supplementary Table 1) and cultured cells for 5 days. Flow cytometry analysis indicated that the CD31^+^CD146^+^ EC population was maintained during SVF expansion, while most of CD45^+^ inflammatory cells were lost (Supplementary Fig. 9a). As our mouse data indicated that inflammatory cells are dispensable for wound revascularization, we used this expansion technique in all subsequent experiments. During ex vivo expansion, the SVF-derived ECs frequently layered on top of the other cell types, forming branched vascular structures (Supplementary Fig. 9b). These structures were not observed when culturing pure ECs, which grew in monolayer (Supplementary Fig. 9c). This observation confirms that human SVF contains a non-endothelial population that promotes the formation of vascular tubes.

**Fig. 5:**
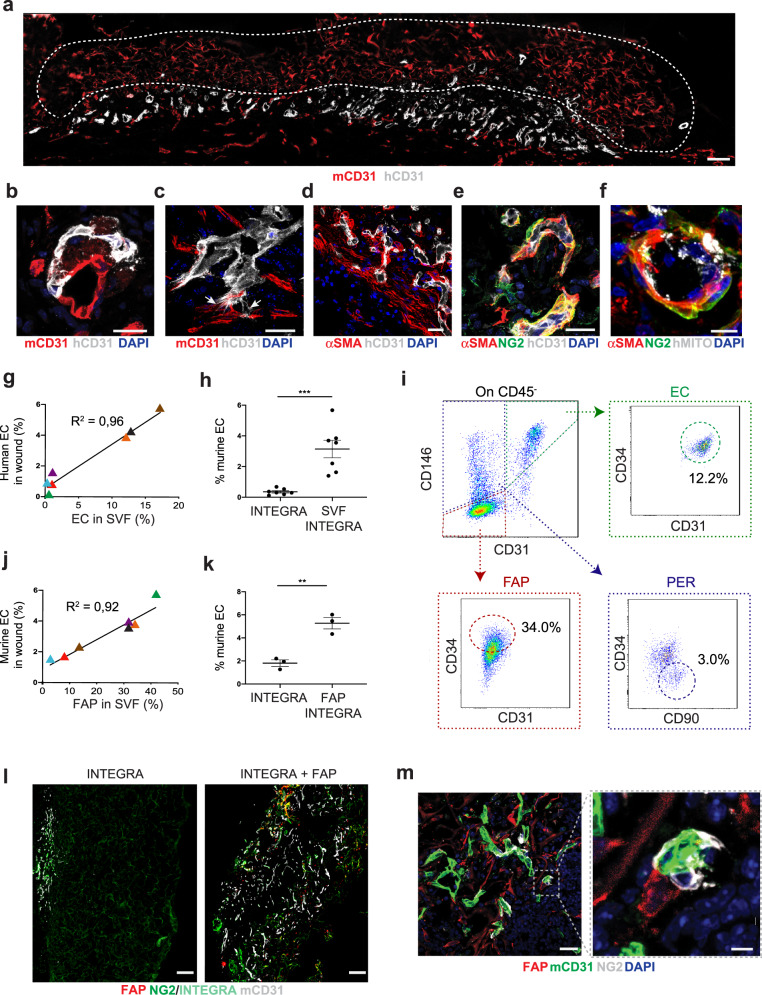
Human SVF induces the revascularization of ischemic wounds through direct and paracrine activities. **a** Representative image of human SVF cells at day 7 after in vivo implantation in ischemic wounds of NSG mice. Human and mouse ECs are stained using species-specific anti-CD31 antibodies. The white, dashed line indicates INTEGRA. **b** Representative image of a hybrid vessel formed by both human (white) and mouse (red) ECs stained with species-specific antibodies. **c** Representative image of a human EC (white) extending numerous filopodia reaching the host murine vasculature (red). A three-dimensional reconstruction of the same field is provided in Supplementary Movie 4. **d** Representative image of the human SVF-derived vascular network (white) wrapped by α-SMA^+^ perivascular cells (red). **e** High-magnification image of a neo-vessel composed of human ECs surrounded by α-SMA^+^NG2^+^ pericytes. **f** High-magnification image of a small vessel surrounded by α-SMA^+^NG2^+^ pericytes of human origin. **g** Coordinate plan showing the correlation between the relative abundance of ECs in the SVF of each donor after ex vivo expansion (*x* axis) and the wound area covered by human ECs, recognized by human-specific anti-CD31 antibodies upon SVF implantation on INTEGRA. Linear regression analysis indicates that high number of ECs results in an extended vascular network of human origin. **h** Quantification of the wound area covered by mouse ECs, recognized by mouse-specific anti-CD31 antibodies, upon implantation of INTEGRA either in the absence or in the presence of human SVF cells. **i** Flow cytometry of human SVF after ex vivo expansion. CD45^−^ cells were plotted according to CD31 and CD146 expression and phenotypically characterized as ECs (CD45^−^CD31^+^CD146^+^CD34^+^, green box), pericytes, PER (CD45^−^CD31^−^CD146^+^CD34^−^CD90^+^, blue box) and fibro-adipogenic progenitors, FAP (CD45^−^CD31^−^CD146^−^CD34^+^CD90^+^, red box). **j** Coordinate plan showing the correlation between the relative abundance of FAPs in the SVF of each donor after ex vivo expansion (x axis) and the wound area covered by murine ECs, recognized by mouse-specific anti-CD31 antibodies upon SVF implantation on INTEGRA. Linear regression analysis indicates that high number of FAPs results in an extended vascular network of mouse origin. **k** Quantification of the wound area covered by murine ECs upon implantation of INTEGRA either in the absence or in the presence of FAPs, sorted from mT mice. **l** Representative images of INTEGRA applied on the wound bed either in the absence (left) or in the presence (right) of mT^+^ FAPs. Colonization by host-derived ECs in the right panel is shown by CD31 staining. **m** Representative image of a wound bed upon application of INTEGRA seeded with mT^+^ FAPs. The inset shows FAPs located in the proximity of NG2^+^ vessels but not directly contributing to pericyte coverage. Data in (**g**, **h**, **j**, **k**) are shown as individual values and SEM (for **h** and **k**); *n* ≥ 3 per group. Scale bar in (**a**, **l**, **m** (left) is 100 μm; in (**b**–**e**) is 25 μm; in (**f**, **m**, right) is 5 μm. **P < 0.01, ***P < 0.001.

To confirm the angiogenic potential of human SVF, we seeded expanded cells on INTEGRA and implanted them on ischemic wounds, generated in NSG mice, as described in Fig. 1a. Like what we observed using murine SVF, human SVF-derived ECs massively colonized the tissue underneath the scaffold and formed a dense, branched vascular network at day 7 after implantation (Fig. 5a). To discriminate between human and mouse ECs, we used species-specific anti-CD31 antibodies and confirmed the presence of hybrid vessels, formed by ECs of both species (Fig. 5b and Supplementary Movie 3). In some cases, we observed human ECs generating fan-shaped filopodia, as they were approaching and contacting murine ECs (Fig. 5c and Supplementary Movie 4). These hybrid vessels were often wrapped by perivascular α-SMA^+^ cells (Fig. 5d). Some of these perivascular cells also expressed NG2, revealing their identity as pericytes (Fig. 5e) and scored positive for human mitochondria-specific antibodies, indicating their human origin (Fig. 5f). Maturation and integration of human cell-derived vessels within the host vasculature was further proven by the presence of a collagen IV^+^ basal membrane, appearing as a continuous line around junction points between human and mouse ECs (Supplementary Fig. 10).

These data indicated a close interaction between ECs and pericytes in the formation of new vessels by the SVF. We confirmed this interaction ex vivo, by purifying both cell types from the SVF and establishing a co-culture, in which we observed EC colonies closely wrapped by pericytes (Supplementary Fig. 11a). Next, we analyzed an existing dataset from single-cell RNA sequencing experiments on human white adipose tissue^18^ to explore the interactome between these two cell types. Briefly, we extracted raw counts of ECs and pericytes from control samples; the resulting dataset included 686 cells (364 ECs and 292 pericytes) and 21,856 annotated genes. Then, we selected 7150 expressed genes, removing those with less than 1 count in all samples and normalized the data (Supplementary Fig. 11b). Finally, we performed the ligand-receptor interaction analysis, obtaining 131 interactions between ECs and pericytes (Supplementary File 1). Among the 81 significant interactions (*P* < 0.01), 56 were ligand-receptor interactions, 20 were ligand–ligand interactions, and 5 were receptor–receptor interactions.

We further selected the most significant interactions known to be relevant for vessel maturation (Supplementary Fig. 11c). To validate the functional role of these ligand-receptor pairs, we silenced both the ligand and the receptor in the corresponding cell type and performed a migration assay to determine whether their absence impaired their capacity to attract each other. The efficiency of silencing by specific siRNAs is reported in Supplementary Fig. 11d. As shown in Supplementary Fig. 11e, f, silencing of PDGFB and its receptor PDGFRB almost completely abrogated the attraction between ECs and pericytes, indicating a major role of EC-derived PDGFB in recruiting PDGFRB-expressing pericytes.

Thus, human SVF stimulates the formation of new vessels, composed of both donor and host cells that integrate into a mature vascular network.

### Human stromal vascular fraction has a dual mechanism: direct contribution of endothelial cells for new vessel formation and contribution of FAPs which exert paracrine activity to support vascularization

To shed light on the role of the various cell types composing the human SVF, we performed flow cytometry on the SVF of the seven donors, according to the gating strategy shown in Supplementary Fig. 12. By linear regression analysis, we could demonstrate a clear relationship between the abundance of ECs gleaned from the SVF of each donor, and the capacity of the isolated SVF to form new blood vessels of human origin (Fig. 5g). In fact, donors could be separated into two groups, depending on the relative abundance of ECs: “high EC” (>12%) and “low EC” (≤1%) (Supplementary Table 1). When we assessed cell engraftment at 7 days by histological analysis, we found that high EC samples generated numerous vessels of human origin, while low EC samples were less able to do so (Supplementary Fig. 13a). Interestingly, some low EC samples (i.e., donor 2) promoted massive expansion of murine vessels (Fig. 5h for quantification and Supplementary Fig. 14 for representative images). Thus, in addition to the direct incorporation of human ECs into vascular structures, the human SVF could exert a strong pro-angiogenic effect on the host vasculature.

We used a panel of surface markers to identify the cell type in the SVF that could account for this paracrine activity. In addition to ECs (CD45^−^CD31^+^CD146^+^CD34^+^), we identified pericytes (CD45^−^CD31^−^CD146^+^CD34^−^CD90^+^ cells) and a large proportion of mesenchymal cells, also named fibro-adipogenic progenitors (FAPs, CD45^−^CD31^−^CD146^−^CD34^+^ cells; Fig. 5i)^19^. As shown in Fig. 5j, the presence of FAPs in the human SVF linearly correlated with the stimulation of host angiogenesis. Also in this case, we identified four donors rich in FAPs (“high FAP”, >30%), which promoted murine vessel formation more efficiently than the three donors who were instead poor in FAPs (“low FAP”, ≤14%) (Supplementary Fig. 13b). Representative images of wounds, colonized by the SVF of each donor and the relative contribution of donor- and host-derived angiogenesis, are shown in Supplementary Fig. 14.

To provide definitive evidence of the paracrine activity exerted by FAPs on the host vasculature, we sorted the FAP population from mT mice according to an established gating strategy (Supplementary Fig. 15a)^20^. By immunofluorescence, we confirmed the purity of sorted FAPs, which were not contaminated by either ECs or hematopoietic cells (Supplementary Fig. 15b). To assess whether FAPs modified the angiogenic phenotype of SVF-derived ECs we established a co-culture. While ECs required a growth factor-rich medium to proliferate and form tubular structure, the presence of FAPs enabled them to keep these angiogenic properties even in a basal medium (Supplementary Fig. 15c–e). Thus, FAPs produce trophic factors for ECs.

We then validated this concept in vivo, by seeding FAPs on INTEGRA, prior to their application on the wound of syngeneic animals. As shown in Fig. 5l, mT^+^ FAPs promoted massive colonization of the scaffold by host vessels, negative for any reporter gene and thus of local origin, in line with the human data. These neo-vessels were largely covered by NG2^+^ pericytes, indicating that FAPs promoted the local expansion of both endothelial and mural cells to form mature vascular structures. However, the FAPs themselves did not acquire any pericyte marker and remained perivascular, consistent with their paracrine activity (Fig. 5m). To identify the secreted factors that could account for this paracrine pro-angiogenic activity, we assessed the expression of more than 50 angiogenic molecules by murine FAPs using a dedicated protein array. Among the most abundant proteins were fibroblast growth factor (FGF)-1, FGF-2, keratinocyte-derived chemokine (KC), monocyte chemoattractant protein (MCP-1), and cellular communication network factor 3 (CCN3), all known to promote blood vessel formation and arterial maturation^21–23^ (Supplementary Fig. 16). For comparison, we ran the same array on SVF-derived ECs and the remaining cells (not ECs, not FAPs) and found that many of these pro-angiogenic molecules were similarly or even more expressed by FAPs than by ECs. Few angiogenic proteins were secreted at low level by other cells in the SVF (Supplementary Fig. 16).

Thus, human SVF promotes wound vascularization by a dual mechanism. On the one hand, SVF-derived ECs directly form new vessels that colonize the scaffold and extend into surrounding tissues. On the other hand, FAPs stimulate the expansion of the host vasculature, which extends into the scaffold, with the eventual appearance of donor-host hybrid vessels.

### Relevance for clinical application of the SVF: functional perfusion, applicability to diabetic patients and scalability

**Fig. 6:**
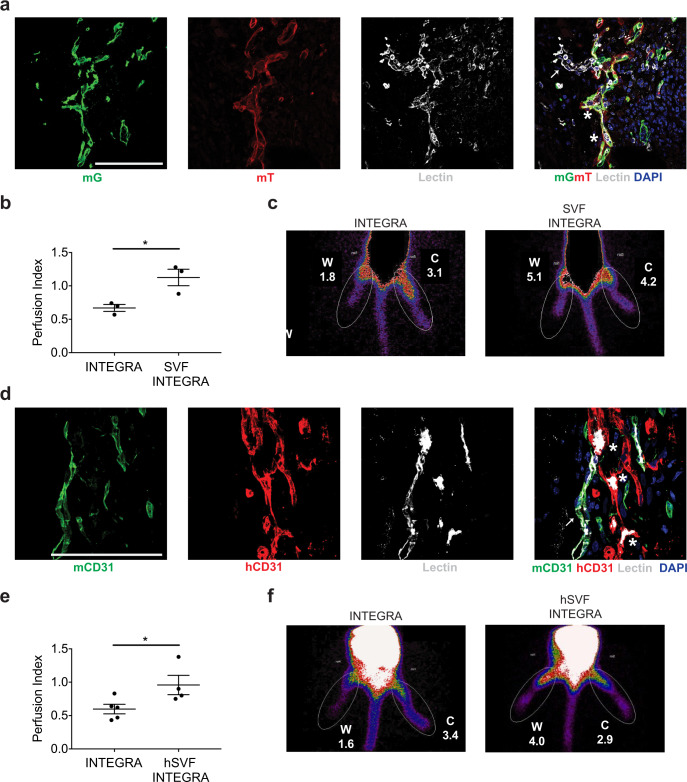
SVF forms functional vessels improving tissue perfusion. **a** Representative image of the vasculature formed by the SVF of Cdh5-CreER;mTmG mice at 7 days, upon perfusion with biotinylated lectin (white). Lectin signal is localized within both donor (mTmG^+^, asterisks) and host (mTmG^−^, arrow) vessels. **b** Quantification of tissue perfusion by scintigraphy at 7 days after implantation of mouse SVF on ischemic wounds in syngeneic mice. Perfusion index is calculated as the ratio of counts within the same ROI applied to both ischemic and nonischemic limb for each animal. **c** Representative scintigraphy images of mice subjected to unilateral hind limb ischemia and wounding, treated with INTEGRA alone or in combination with syngeneic SVF cells. Radioactive counts within the ROI are indicated for both wounded (W) and control (C) limbs. **d** Representative image of the vasculature formed by human SVF at 7 days after its implantation in NSG mice, upon perfusion with biotinylated lectin (white). Lectin signal is localized within both donor (red, asterisks) and host (green, arrow) vessels. **e** Quantification of tissue perfusion by scintigraphy at 7 days after implantation of human SVF on ischemic wounds in NSG mice. Perfusion index is calculated as the ratio of counts within the same ROI applied to the ischemic and control (nonischemic) limb for each animal. **f** Representative scintigraphy images of NSG mice subjected to unilateral hind limb ischemia and wounding, treated with INTEGRA alone or in combination with human SVF cells. Counts within the ROI are indicated for both wounded (W) and control (C) nonischemic limbs. Data in (**b**, **e**) are shown as mean ± SEM. *n* ≥ 3 per group. Scale bar in (**a**, **d**) is 100 μm. *P < 0.05.

To validate the functionality of SVF-induced vessels, we perfused animals intravenously with biotinylated lectin to label the luminal side of ECs reached by the blood flow, as shown in Supplementary Fig. 17a. We first applied this strategy to the mouse-in-mouse model, in which the implanted SVF was derived from Cdh5-CreER/mTmG mice. As shown in Fig. 6a and Supplementary Movie 5, numerous vessels formed by donor cells were labeled by lectin, indicating their actual perfusion. From these vessels, some lectin^+^ sprouts emerged, but did not contain fluorescently labeled ECs, confirming again the formation of mosaic networks between donor and host ECs.

We also quantified the perfusion of treated limbs by scintigraphy with pinhole collimator. Tissue perfusion was calculated as the ratio between radioactive counts within an equivalent region of interest (ROI) in the wounded leg (right) and in the control leg (left) of each animal. As shown by the quantification and representative images in Fig. 6b, c SVF application significantly improved tissue perfusion. Consistent results were obtained by assessing wound perfusion by laser doppler imaging (Supplementary Fig. 17b).

Finally, we evaluated the functional competence of human SVF-derived vessels by systemic lectin injection and confirmed the presence of lectin signal within vessels of both human and mouse origin, which were often interconnected, forming hybrid structures (Fig. 6d and Supplementary Movie 6). Assessment of tissue perfusion by scintigraphy confirmed the functionality of human SVF-derived neo-vessels (Fig. 6e for quantification and Fig. 6f for representative images).

**Fig. 7:**
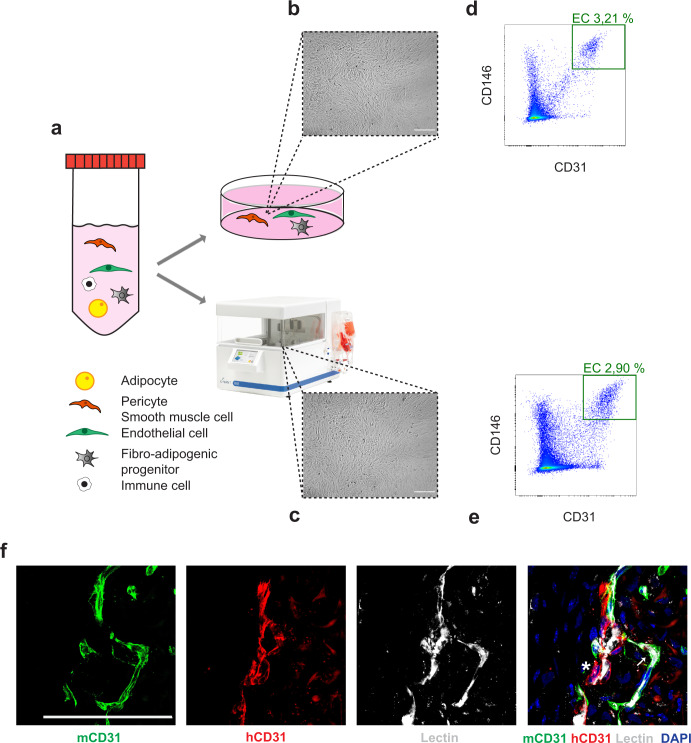
Expansion of diabetic, human SVF using an automated bioreactor. **a** Schematic representation of the different cell types composing the human SVF. **b** Bright-field microscopy of SVF cells expanded for 7 days in a standard culture dish. **c** Bright-field microscopy of SVF cells expanded for 7 days using a bioreactor. **d** Flow cytometry profile of cells expanded for 7 days in a standard culture dish. **e** Flow cytometry profile of cells expanded for 7 days using a bioreactor. The number of ECs is comparable to panel **d**. **f** Representative images of the vasculature formed by diabetic, human SVF at 7 days after its implantation in NSG mice, upon perfusion with biotinylated lectin (white). Lectin signal is localized within both donor (red, asterisk) and host (green, arrow) vessels. Scale bar in (**b**, **c**, **f**) is 100 μm.

As nonhealing wounds primarily affect diabetic patients, we sought to validate the therapeutic potential of the SVF derived from individuals affected by overt diabetes. Because human cell therapy would benefit from an automatic and standardized cell culture procedure, we cultured human SVF from four diabetic patients either in static conditions or using an automated, sterile bioreactor, as schematically represented in Fig. 7. Microscopic examination at 7 days revealed very similar cell morphology in both culture conditions (shown for one representative patient in Fig. 7a–c). In addition, flow cytometry revealed a comparable composition of the SVF expanded in the bioreactor and in traditional static culture (Fig. 7d, e; see Supplementary Fig. 12 for gating strategy). This validates the feasibility of large-scale SVF expansion within a sterile environment, which can be performed in automated bioreactors, housed in hospitals, streamlining the therapeutic application.

Finally, we assessed the functional competence of the diabetic SVF expanded in the bioreactor in contributing to neo-vessel formation in our model of ischemic wounds in NSG mice. As shown in Supplementary Fig. 18, we again observed the formation of hybrid vessels between human SVF cells and mouse ECs. These hybrid vessels were functional and connected with the host vasculature, as shown by lectin perfusion (Fig. 7f).

These results prove that the SVF can be expanded from diabetic patients using a bioreactor and maintains its capacity to form functional vessels when transplanted into an ischemic wound.

## Discussion

This study has important clinical implications. Having shed light on the mechanisms of action of the SVF, it represents a steppingstone toward the reproducible and effective adoption of the SVF as standard care for nonhealing wounds.

We characterized the major cell populations that compose both mouse and human SVF and provided the first evidence that both ECs and pericytes in the SVF directly contribute to the formation of a hierarchical vascular network composed of hybrid donor-host vessels that are functionally connected with the host circulation and eventually result in improved perfusion of the ischemic wound. At the same time, FAPs in the SVF exert a paracrine angiogenic activity on resident ECs, further improving wound revascularization.

A major feature of novelty in our work is the generation of a mouse model of nonhealing wound, which recreates the clinical condition by inducing a full-thickness skin lesion in ischemic hindlimbs. Evaluation of local therapies for wound healing has so far relied on the generation of a skin wound on the mouse dorsum, without reproducing the conditions that hamper wound healing in humans. While diabetes is a major co-morbidity responsible for nonhealing wounds, existing murine models fail to mimic the human disease, due to their acute phenotype leading to uncontrollable hyperglycemia and premature death, in the absence of chronic complications^24,25^. Because both diabetes and peripheral artery disease often coexist and eventually result in tissue ischemia^26,27^, we first induced severe hindlimb ischemia by the excision of a proximal segment of the femoral artery and then generated a full-thickness skin wound, to mirror the vascular deficit that characterizes nonhealing wounds in patients. This represents the first murine model of a cutaneous ischemic ulcer, like what has been attempted in pigs and rabbits^24^. Some limitations remain, largely related to the different modalities by which wounds heal in humans and mice. While human wounds mainly rely on re-epithelization and, in ischemic conditions, do not heal, murine wounds close by contraction, resulting in complete healing in a few weeks, also depending on mouse strain^28^. Most of hindlimb ischemia studies have been performed on Balb/c mice, as this strain develops the highest level of ischemia^29–32^. In our study, we had to use also NSG and C57BL/6 mice, despite their higher tendency to spontaneous reperfusion, to be able to implant human and genetically labeled SVF cells, respectively.

In our work, we have demonstrated the efficacy of the SVF in two models. First, we used a mouse-in-mouse approach, harvesting the SVF from transgenic mice to trace the fate of both endothelial and non-endothelial cells. Second, our human-in-mouse model entailed the isolation of human SVF and its transplantation into NSG immunodeficient mice, in which we could identify transplanted ECs using species-specific antibodies. In both models, SVF-derived ECs form new blood vessels, in accordance with previous evidence^33^, composed of arteries, veins and capillaries, resembling the vasculature of healthy skin. Further, we showed that these vessels were hybrid, composed of both donor and host cells, and surrounded by SVF-derived smooth muscle cells and pericytes, which are essential for their structural and functional maturation, as well as to avoid vessel regression^21,34^. We observed both host perivascular cells wrapped around SVF-derived endothelial tubes, and vice versa, confirming multiple intercellular interactions between transplanted and resident cells.

In addition to blood vessels, SVF cells contributed to the formation of lymphatics. While recent data suggest some plasticity between lymphatic and blood ECs^35,36^, our data using Apln-Cre reporter mice demonstrate that angiogenesis and lymphangiogenesis are driven by Apln^+^ and Apln^−^ cells, respectively, in line with previous observations in the ischemic heart and in cancer^35,37^. Whether the formation of new lymphatics is relevant in the therapeutic efficacy of the SVF, by dampening skin inflammation^38^, remains a plausible, yet unproven hypothesis.

A few studies have investigated the potential of human, adipose-derived ECs to form new vessels^39,40^, without proving their integration with the host vasculature and overall improvement in tissue perfusion. These studies gave inconclusive results, which have so far prevented the incorporation of the SVF in routine clinical practice. A few outstanding questions need to be addressed to adopt the SVF as a standard care^13,41^. For instance, which cell types in the SVF are essential and what is their specific role? Can we predict therapeutic efficacy based on SVF composition? Our work has shed light on these relevant questions.

By using a panel of validated markers^42–44^, we have identified four major cell populations within the SVF: immune cells, ECs, pericytes and FAPs. Immune cells, which compose up to 50% of freshly isolated SVF, seem to be dispensable, as their depletion did not interfere with new blood vessel formation. This is consistent with our evidence that human SVF is still active upon ex vivo expansion, which results in the loss of immune cells, as well as with previous data, showing that host- and not donor-derived macrophages are required for the therapeutic efficacy of the SVF^33^.

Unlike immune cells, ECs are directly incorporated into the newly formed vessels and their depletion abolished the formation of any donor-derived vessel. Our genetic tracing system indicated that SVF-derived pericytes also directly contributed to neo-vessel formation, as numerous SVF-derived perivascular cells wrapped around ECs of both donor and host origin, consistent with the capacity of other mesenchymal cells to improve EC engraftment and angiogenic potential^45^. In contrast, the transplantation of purified ECs, in the absence of pericytes, generated immature vessels, deprived of perivascular cells. By bioinformatic analysis we identified 55 potential interactions between SVF-derived ECs and pericytes, and validated the function of five ligand-receptor pairs, previously associated with vessel maturation^34^. PDGFB was the most effective factor produced by ECs, capable of recruiting PDGFRB^+^ pericytes, in line with previous evidence showing a role for PDGFB/PDGFRB^+^ signaling in mediating the paracrine activity of ECs in both mesenchymal cell engraftment^46^ and pericyte recruitment in other organs, such as brain, liver and cancer^47–49^.

Finally, we characterized the property of FAPs to promote host angiogenesis through the secretion of a pool of pro-angiogenic factors, as shown by our protein array and previous evidence in regenerating muscle^19,50^.

Collectively, these findings provide a steppingstone toward the reproducible and effective adoption of the SVF as a standard care for nonhealing wounds, possibly in combination with existing therapies, such as platelet-rich plasma (PRP)^51^ or hair follicle-derived epidermal substitutes^52,53^, which provide growth factors and epidermal cells, respectively, but not contain vascular cells. We propose that a minimal abundance of both ECs and FAPs is required to ensure a significant increase in the number of new, functional vessels, which could set the stage for the definition of quality standards in SVF cell therapy release testing. In addition, we successfully isolated the SVF from diabetic patients and showed that it retains the capacity to form new vessels, functionally connected with the host circulatory system. Finally, we scaled up and standardized SVF expansion using an automated bioreactor, designed to be used as intramural care within hospitals, in a sterile manner and without the need of either clean rooms or transport of the autologous tissue for expansion.

## Methods

### Isolation and culture of mouse and human stromal vascular fraction (SVF)

Mouse SVF was isolated from inguinal white adipose tissue of adult BALB/C (Envigo), mTmG (The Jackson Laboratory, 007576), Cdh5-CreER/mTmG^54^ or Apln-CreER/mTmG mice^17^. Human SVF was isolated from the subcutaneous lipoaspirate of thigh, abdomen, or hip. The SVF of diabetic patients was isolated from the abdominal subcutaneous adipose tissue. The collection and manipulation of human samples was performed in compliance with all relevant ethical regulations, including the Declaration of Helsinki, upon approval by the Regional Ethics Committee of Friuli Venezia Giulia, Italy (authorization n. 15569/P/GEN/ARCS) and obtainment of written informed consent by all patients.

The adipose tissue was rinsed with calcium- and bicarbonate-free Hank’s solution with HEPES (CBFHH) and minced using fine scissors until the tissue suspension appeared homogeneous. The tissue was transferred in gentleMACS C Tubes (Miltenyi Biotec, 130-093-237, 130-096-334) containing a pre-warmed digestion solution composed of 1 mg/mL Collagenase NB4 Standard Grade (Nordmark Biochemicals, S1745401), 0.5 mg/mL DNase II (Sigma, D8764) and 100 μg/mL antibiotics (gentamicin, penicillin/streptomycin) dissolved in BFHH (Bicarbonate-Free Hanks' solution with HEPES) supplemented with 2 mM CaCl_2_. GentleMACS™ Octo Dissociator with Heaters (Miltenyi Biotec, 130-096-427) was used to digest the tissue running the program *37C_mr_ATDK_1*. The digested tissue was then diluted in Dulbecco’s Modified Eagle Medium (DMEM, Sigma) supplemented with 10% fetal bovine serum (FBS) and centrifuged at 700 × *g* for 10 min. After removing the oily and liquid layers, the pellet was resuspended in DMEM with 10% FBS and filtered using a 700-μm pore size cell strainer (Falcon, 352350). The cell suspension was centrifuged at 500 × *g* for 8 min. Finally, SVF cells were resuspended in Endothelial Cell Growth Medium-2 (EGM-2, Lonza, CC-3162).

Mouse ECs were positively selected from the SVF by magnetic separation using Dynabeads® Sheep anti-Rat IgG (ThermoFisher, 65305) in combination with primary rat IgG anti-mouse CD31 (BD Pharmingen, 557355) following the manufacturer’s instructions. To assess the trophic support of FAPs on ECs, 3 × 10^4^ SVF-derived CD31^+^ cells were plated in primary 96-well plates coated with fibronectin/gelatin using either endothelial basal medium (EBM)-2 or EGM-2. FAPs were then plated together with CD31^+^ cells at a 1:1 ratio in EBM-2 and kept in culture for 5 days.

Mouse ECs were depleted from the SVF by magnetic separation using Dynabeads® as described above. EC-depleted SVF was centrifuged at 500 × *g* for 8 min, and the pellet was resuspended in EGM-2 for in vivo application.

Mouse immune cells were depleted from the SVF using mouse CD45 MicroBeads (Miltenyi Biotec, 130-052-301) following the manufacturer’s instructions. After the negative selection, the CD45^−^ cells were centrifuged at 500 × *g* for 8 min. The pellet was then resuspended in EGM-2 for in vivo application.

Mouse pericytes were positively selected from the SVF by magnetic separation using Dynabeads® M-270 Streptavidin (ThermoFisher, 11035) in combination with goat polyclonal biotinylated antibody anti-mouse PDGFR-beta (R&D/Bio-Techne, BAF1042) following the manufacturer’s instructions^55^.

SVF cells were centrifuged at 500 × *g* for 5 min and resuspended in FACS buffer (PBD, 2 mM EDTA, 2% FBS). Cells were first incubated with murine Fc blocking antibody (dilution 1:100, Biolegend, 101320) for 10 min at room temperature in FACS buffer. Later, they were centrifuged and incubated with staining cocktail for 25 min on ice protected from light. The staining cocktail contained: PE anti‐CD45 (dilution 1:200, Biolegend, 103105), BV421 anti‐CD31 (dilution 1:200, BD Biosciences, 562939), PECy7 anti‐Sca1 (dilution 1:2000, Invitrogen, 25-5981-81). After incubation, cells were rinsed twice, resuspended in FACS buffer and sorted using Aria cytometer (FACS ARIA II BD). To confirm the purity of the sorted FAP population, 3 × 10^4^ FAPs were plated in primary 96-well plates coated with fibronectin/gelatin in EGM-2 for 24 h.

The isolated SVF was plated at the density of 5.2 × 10^4^ cells/cm^2^ in primary plates coated with fibronectin/gelatin for 5 days in EGM-2. Cells were detached and collected using TrypLE™ Select CTS™ (A1285901, ThermoFischer). A similar protocol was used to expand the human SVF in the bioreactor NANT 001 (VivaBioCell).

### Angiogenic protein array on purified SVF sub-populations

CD45^−^CD31^−^ SVF cells, CD31^+^cells, and FAPs were purified from the adult mouse SVF as described. The expression profile of angiogenesis-related proteins was analyzed using Proteome Profiler Mouse Angiogenesis Array Kit (R&D/Bio-Techne, ARY015) as per the manufacturer’s instructions. The membranes were incubated with SuperSignal West Dura kit (Thermo Scientific, 34577) and images were acquired with BioRad ChemiDoc Touch. Pixel density was quantified using ImageJ/Fiji software (NIH).

### Gene silencing in EC-pericyte co-culture

To test the crosstalk between SVF-derived ECs and pericytes, we set up a migration assay by placing a two well silicone insert with a defined cell-free gap (Ibidi, 80209) into 24-well plates coated with fibronectin/gelatin. Each cell type was then seeded at the density of 1.5 × 10^4^ cells in one of two halves of the well. The day after, cells were transfected with 50 nM siRNA (Dharmacon- Horizon, siGENOME Smart pool siRNA) using RNAiMAX (Invitrogen, 13778-150) according to the manufacturer’s instructions, as indicated in Supplementary Table 2. The silicon inserts were removed 72 h after transfection and fresh EBM-2 was added to the culture. Cells were fixed using 4% PFA after an additional 16 h.

### Mouse model of ischemic, nonhealing wound

Animals were housed in compliance with institutional guidelines, national and international laws and policies. All experimental procedures were approved by the ICGEB Animal Welfare Board, as required by the EU Directive 2010/63/EU, and by the Italian Ministry of Health (authorization n. 213/2022-PR).

To activate Cre-mediated recombination, 4-hydroxytamoxifen (Sigma, H6278) was dissolved in corn oil (20 mg/ml) and injected intraperitoneally (20 mg/Kg) into donor Cdh5-CreER/mTmG mice 7 days before SVF isolation. Syngeneic recipient mice were similarly treated with Tamoxifen at 1 and 2 days after implantation of the SVF derived from Apln-CreER/mTmG SVF.

Surgical induction of unilateral hind limb ischemia was performed in adult BALB/C (8 weeks old) for perfusion experiments, C57BL/6 (48 weeks old) for genetically labeled cell transplantation and NSG (48 weeks old) mice for human cell transplantation. General anesthesia was induced by intraperitoneal injection of ketamine 40 mg/kg (Imalgene 1000)/xylazine 100 mg/kg (Sigma). Mice were placed in a supine position on a heated plate, with the left limb extended and immobilized. An incision of the skin, ~1.5 cm long, was made at the level of the knee towards the medial thigh under dissection microscope. The femoral artery was separated from the femoral vein and nerve and occluded using a 7-0 silk suture at two sites: close to the inguinal ligament (proximal ligation) and under the knee (distal ligation). The segment between distal and proximal ligations was eventually excised. After having sutured the skin incision, mice were placed in a prone position and a full-thickness skin lesion was induced at the level of the posterior femoralis biceps muscle using a disposable punch for skin biopsies (6 mm in diameter). Animals were kept on the heated plate in a recovery cage and monitored until awake.

SVF cells (5 × 10^5^) were either injected into the subcutaneous tissue surrounding the wound perimeter or seeded on INTEGRA Dermal Regeneration Template with silicone layer (Integra Life Science Corporation) in EGM-2 for 1 h at 37 °C and 5% pCO_2_. The INTEGRA scaffold (6 mm × 5 mm) seeded with SVF cells was directly applied to the wound and sutured using a 5-0 wire.

The area of each wound was quantified at multiple time points, using a caliper to measure is greatest length and the perpendicular greatest width. The two values were multiplied to estimate wound area and their product normalized on its initial value.

Biotinylated lectin (VectorLab/DBA, B-1175-1, 5 μg) was intravenously injected 10 min prior to animal sacrifice.

For laser doppler perfusion imaging, mice were anesthetized via 1.5% isoflurane under constant oxygen and placed in a prone position. The wound area was scanned (PeriCam PSI, PeriMed) and mean perfusion was used to calculate the relative perfusion ratio (ischemic/nonischemic).

Perfusion scintigraphy with pinhole collimator was performed under general anesthesia as described before. Either BALB/C or NSG mice were anesthetized and intravenously injected with 3.7 mBq of 99mTc-Tetrofosmin (MioViewTM GE Healthcare). After 10 min, mice were placed in a prone position with both ischemic left limb and nonischemic right limb extended and immobilized. Images were acquired for 10 min using a gamma camera equipped with a pinhole collimator (Siemens Ecam)^30^.

### Immunofluorescence

For histological analysis, mice were systemically perfused with 10 ml of 1% v/v PFA (Santa Cruz) and PBS. The INTEGRA scaffold and tissue underneath were collected and fixed overnight at 4 °C in 2% PFA. Tissues were equilibrated in 30% sucrose overnight at 4 °C before embedding in OCT (Bio-Optica). Sections were permeabilized with 0,5% v/v Triton X-100 (Sigma-Aldrich) in PBS for 20 min and blocked in 5% BSA (bovine serum albumin—Roche) in PBS for 1 h. The samples were incubated overnight at 4 °C with the primary antibody diluted 1:100 in 1% BSA, 0.1% Tween-20 (Sigma-Aldrich) in PBS. Subsequently, secondary antibody was diluted 1:500 in 1% BSA, 0,1% Tween-20 in PBS for 2 h at room temperature. Nuclei were counterstained with Hoechst 33342 (Invitrogen, H3570) diluted 1:5000 in PBS. Finally, slides were mounted using Mowiol mounting medium (Sigma-Aldrich).

The same protocol was performed on cells that were previously fixed with 4% PFA for 20 min and permeabilized with 0.5% Triton X-100 (Sigma) in PBS for 2 min.

### Microscopy and image analysis

Images were acquired with a Nikon Eclipse Ti-E inverted fluorescent microscope equipped with DC- 152Q-C00-FI using NIS V4.30 software (Nikon) and a ZEISS LSM 880 with Airyscan.

At least four images per sample were acquired for both ex vivo and in vivo experiments. Images were analyzed using ImageJ2 (Fiji) software. Z projection was performed on maximum intensity. Stitched images were acquired using NIS V4.30 software (Nikon). The analysis of host and donor vasculature was limited to the central area of each stitch (5120 × 2160 pixels).

### Flow cytometry

Cells were centrifuged at 500 × *g* for 5 min and resuspended in FACS buffer with human Fc blocking antibody (dilution 1:100, BD Pharmigen, 564219). After 10 min, the cells were centrifuged at 500 × *g* for 5 min and resuspended in staining cocktail for 25 min on ice, protected from light. The staining cocktail contained: CD34 PE (1:500, clone 4H11, Invitrogen, 12-0349-42), CD146 Ax647 (1:500, clone P1H12, Biolegend, 361013), CD45 BV421 (1:500, clone H130, BD Horizon, 563880), CD31 FITC (1:500, clone WM59, Invitrogen 11-039-42), CD90 BV786 (1:500, clone 5E10, BD OptiBuild, 740986). After incubation, cells were rinsed twice with FACS buffer and fixed in 4% PFA for acquisition with Influx cytometer (FACS Celesta BD).

### Interactome analysis

The interactome was generated from the public available dataset of Hildreth et al.^18^ (GEO accession: GSE155960) to collect scRNA-Seq data of Smooth Muscle Cells (*n* = 292) and ECs (*n* = 394). The “DaMiRseq” R package was used to select expressed genes and to perform the log2CPM normalization and the exploratory analysis^56^. Ligand-receptor interactions were identified by CellPhoneDB (v.2.1.7)^57^.

### RNA extraction and real-time PCR quantification

Cells were lysed using TRIzol (Invitrogen, 15596018) and total RNA was extracted following the manufacturer’s instructions. Total RNA (400 ng) was retrotranscribed using First Strand cDNA Synthesis Kit (Thermo Scientific, K1612) with random hexameric primers. Gene expression was quantified by real-time PCR using SYBR Green (GoTaq® qPCR Master Mix, Promega, A6002) and primers indicated in Supplementary Table 5. *Gapdh* was used as a housekeeping gene.

### Statistical analysis

Data are represented as mean ± standard error of the mean (SEM) and the sample number is indicated in each figure legend. Data were obtained from at least three independent experiments. The significance of differences was assessed using the GraphPad Prism 8 software. The normal distribution of data sets was tested and, depending on the results, multiple comparisons were performed with the parametric one- or two-way analysis of variance (ANOVA) or with the nonparametric Kruskal–Wallis test, while single comparisons were analyzed with the nonparametric Mann–Whitney test or the parametric one-tailed *t* test. Results with *P* values of less than 0.05 were considered statistically significant (*P < 0.05, **P < 0.01, ***P < 0.001, ****P < 0.0001).

### Reporting summary

Further information on research design is available in the Nature Research Reporting Summary linked to this article.

## Supplementary information

Supplementary Information

Supplementary Data 1

Supplementary movie 1

Supplementary movie 2

Supplementary movie 3

Supplementary movie 4

Supplementary movie 5

Supplementary movie 6

Reporting Summary

## Data Availability

Most data generated or analyzed during this study are included in this published article (and its supplementary information files). Raw data are accessible as Supplementary Information (Supplementary Data 1 file).
