# Supplementary material for: Ischemic wound revascularization by the stromal vascular fraction relies on host-donor hybrid vessels
Source: NPJ Regen Med. 2023 Feb 11;8:8. doi: 10.1038/s41536-023-00283-6 (PMC9922297; doi:10.1038/s41536-023-00283-6)
Supplement: Supplementary file 1 — Supplementary Information [file 41536_2023_283_MOESM1_ESM.pdf]

Supplementary information

Supplementary Figures

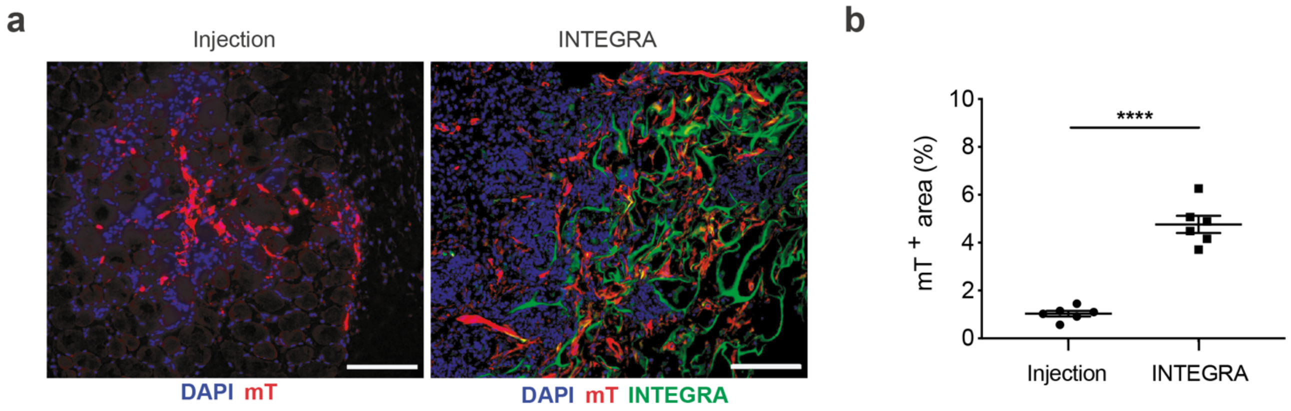

Supplementary Figure 1. SVF seeding on INTEGRA increases cell engraftment in ischemic wounds

a) Representative images of mT^+ SVF cells either injected in the subcutaneous tissue surrounding the wound perimeter (left panels) or applied to an INTEGRA scaffold prior to wound dressing (right panels) at day 3 after cell implantation. Scale bar, 100 µm

b) Quantification of the area occupied by the mT^+ SVF cells in the conditions described in panel a. Data are shown as mean ± S.E.M. n = 6 per group. Statistical significance was determined using unpaired Student's t-test. ****$P < 0.0001$.

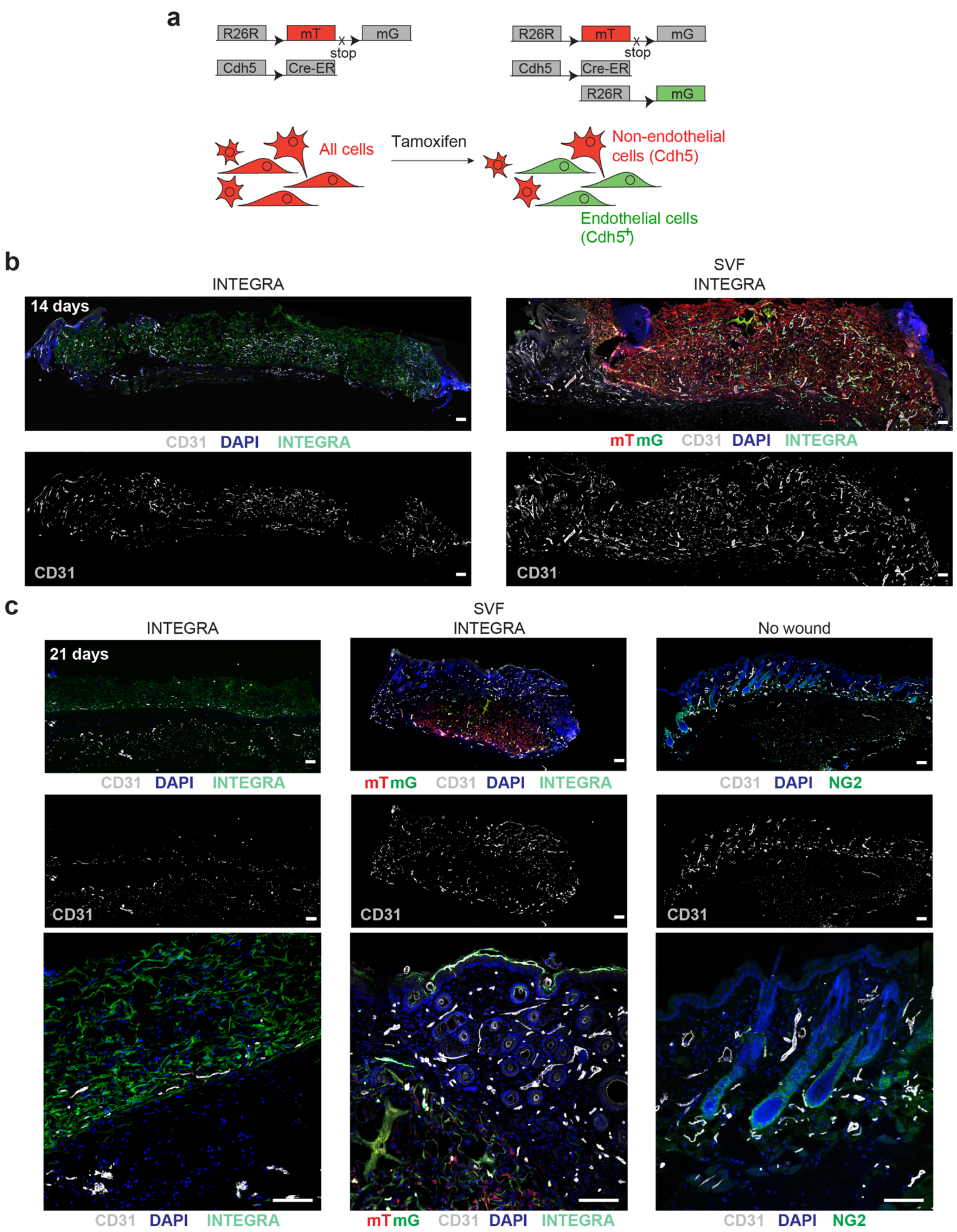

Supplementary Figure 2. Genetic tracing of SVF cells over time

a) Schematic representation of Cdh5-CreER;mT/mG mouse reporter system. The SVF is purified after 5 days of tamoxifen injection, to have all cells labelled in red by mT and ECs specifically labelled in green by mG.

b) Representative images of $CD31^+$ vessels within INTEGRA scaffolds at 14 days after implantation of Cdh5-CreER;mTmG SVF cells.
c) Representative images of $CD31^+$ vessels within INTEGRA scaffolds at 21 days after implantation of Cdh5-CreER;mTmG SVF cells. Panels on the right represent a healthy skin stained for NG2 to label hair follicles.

Scale bar in b and c, 100 µm

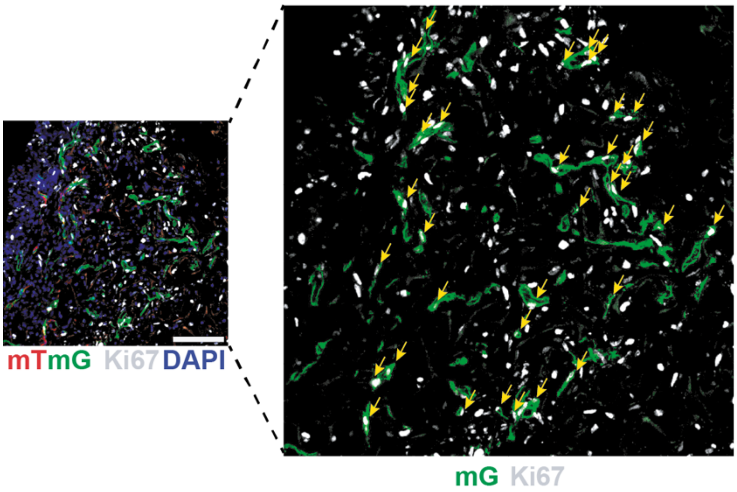

Supplementary Figure 3. SVF-derived ECs proliferate *in vivo*

Immunofluorescence staining of an INTEGRA scaffold populated by Cdh5-CreER;mTmG SVF cells at day 7 after implantation. Yellow arrows indicate Ki67^{+} proliferating ECs.

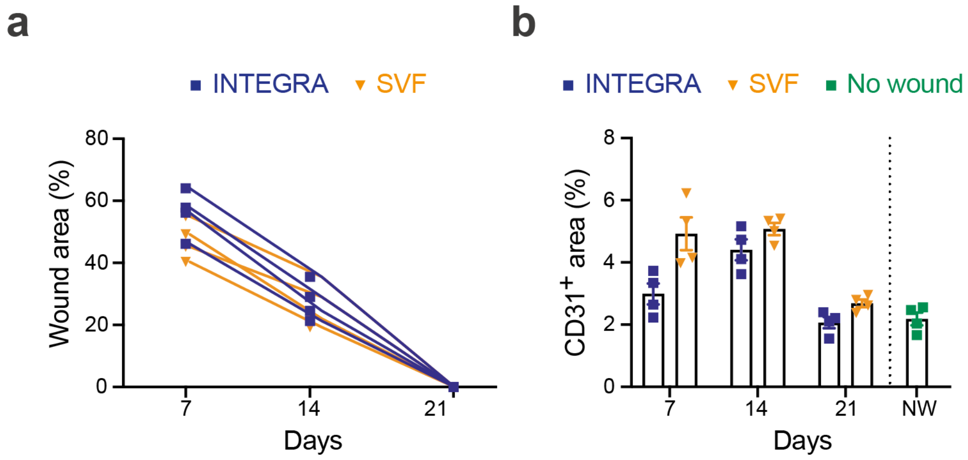

Supplementary

Supplementary Figure 4. SVF effect on normoxic wounds

a) Quantification of normoxic wound area upon application of INTEGRA scaffold, either alone or in combination with SVF cells, at the indicated time points. Data are shown as individual values.

b) Quantification of $CD31^+$ area in normoxic wounds upon application of INTEGRA scaffold, either alone or in combination with SVF cells, at the indicated time points. Data are compared to those of a healthy skin. Data are shown as mean ± S.E.M. n = 4 per group. Statistical significance was determined using two-way ANOVA for repeated measurements

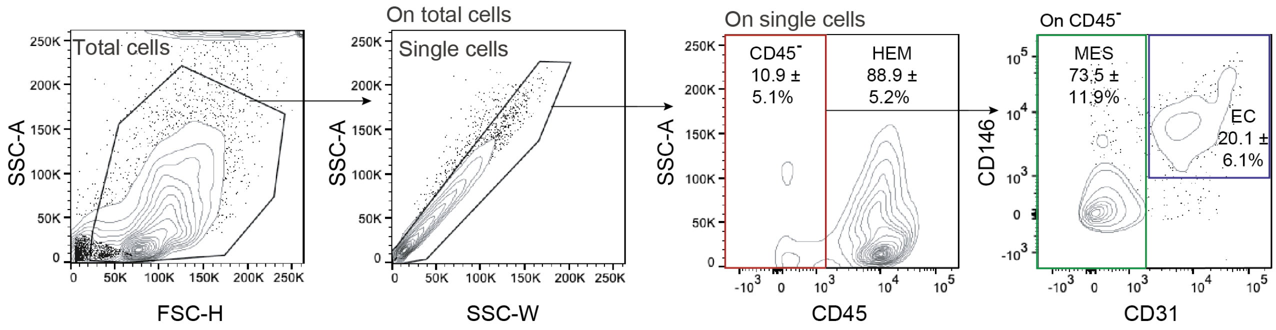

Supplementary Figure 5. Characterization of mouse SVF by flow cytometry

Representative dot plots depicting the gating strategy based on physical parameters to exclude cellular debris and doublets. $CD45^-$ cells (red box) were plotted according to CD31 and CD146 expression to phenotypically characterize hematopoietic cells (HEM, $CD45^+$), ECs ($CD45^-$ $CD31^+CD146^+$, blue box), and mesenchymal cells (MES, $CD45^-CD31^-$, green box). Data are shown as mean ± S.E.M. n = 5 per group.

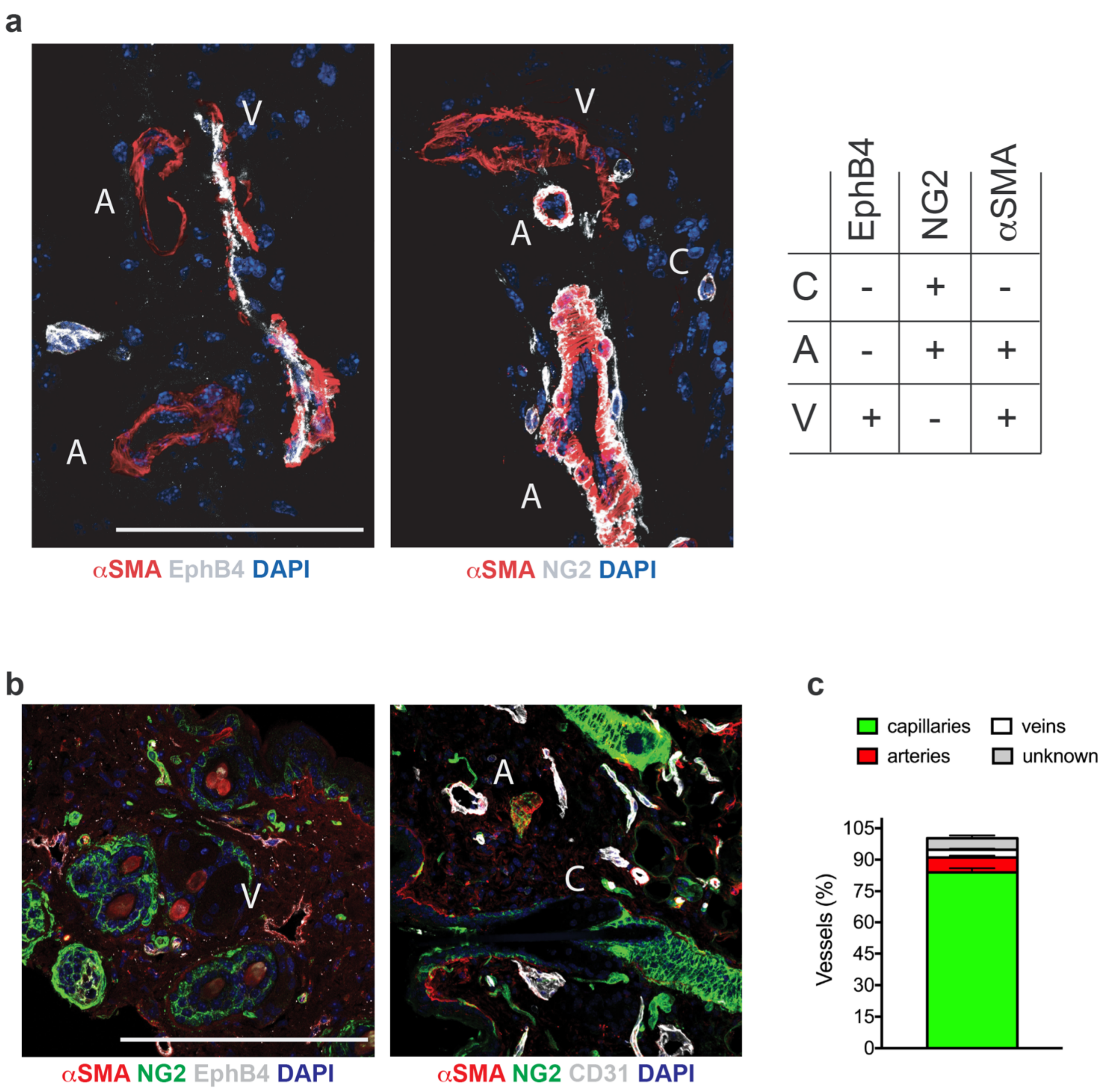

Supplementary Figure 6. Immunophenotyping of vessel types.

a) The three major vessel types composing any vascular network, namely arteries (A), veins (V) and capillaries (C), are identified by a combined immunostaining for α-SMA, NG2 and EphB4, as detailed in the matrix on the right.

b) Representative image of healthy mouse skin stained for the indicated markers to visualize the various vessel types, as identified in panel A.

c) Quantitative analysis of the different vessel types composing the mouse healthy skin.

Scale bar in a, b, 100 µm.

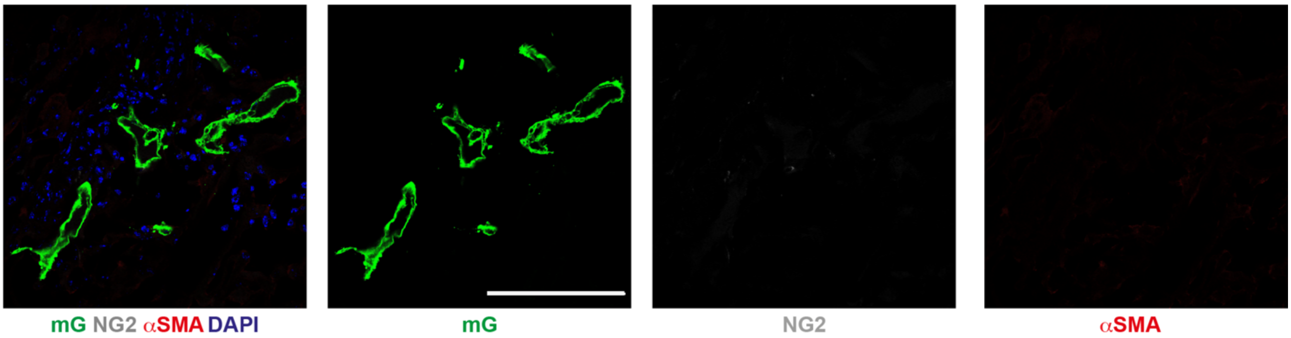

Supplementary Figure 7. Vessels formed by pure ECs are structurally immature.
Representative image of a wound bed covered by INTEGRA seeded with purified ECs from Cdh5-CreER/mTmG mice, treated with tamoxifen for 7 days, stained for the perivascular cell markers α-SMA and NG2. Scale bar, 100 μm.

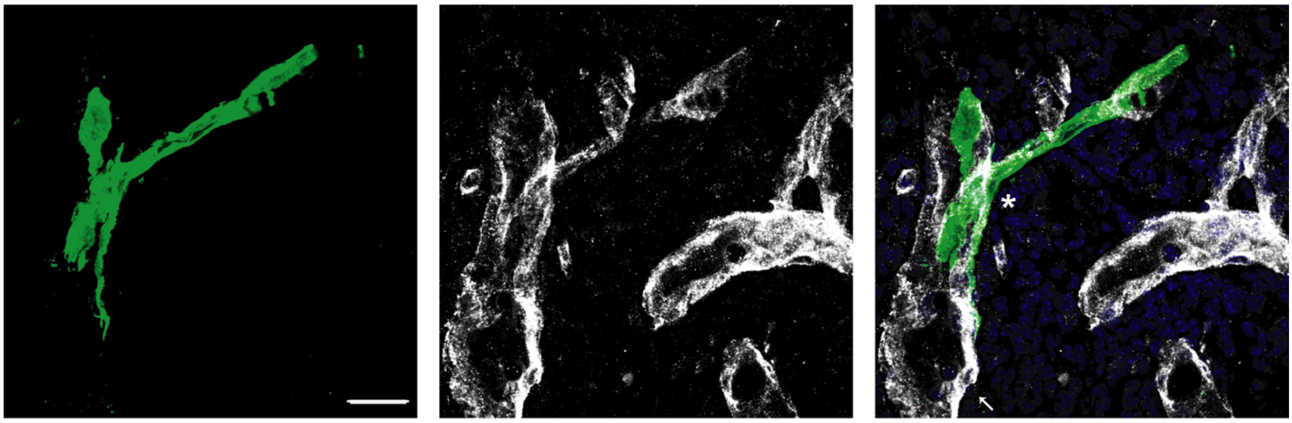

Supplementary Figure 8. Hybrid vessels formed by both SVF-derived and host ECs.
Representative image of a venous network labeled in white by Eph4, composed of both SVF-derived mG^+ ECs (asterisk) and host mG^- EC (arrow). Scale bar, 25 µm.

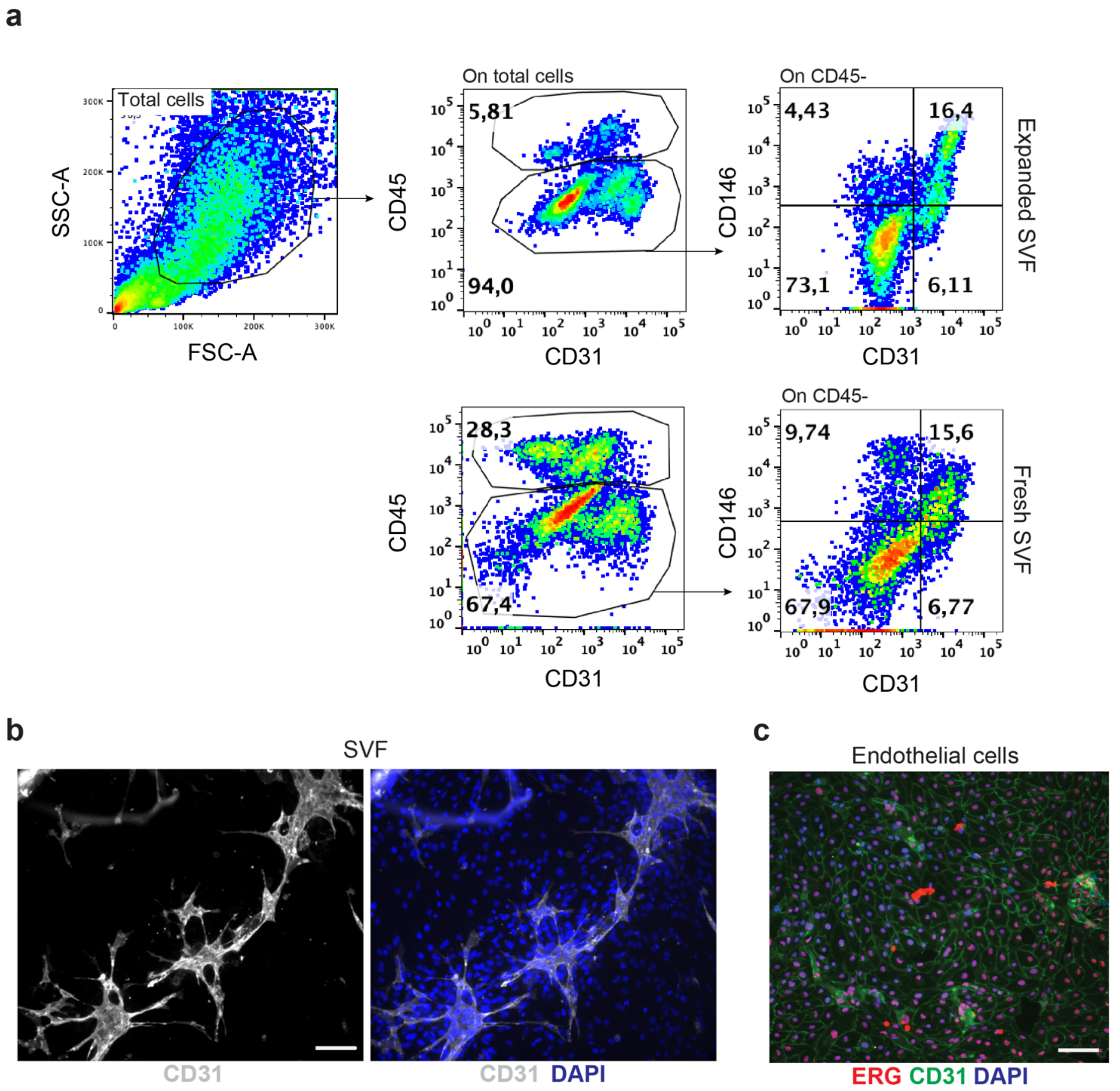

Supplementary Figure 9. Human SVF phenotyping.

a) Flow cytometry plots showing the gating strategy and the different populations composing the SVF either after five days of *ex vivo* expansion (upper panels) or immediately after harvesting (lower panels).

b) Representative image of human SVF in culture, with ECs (labeled by CD31) forming tubular structures over the other cell types

c) Representative image of a pure EC culture (labeled by ERG and CD31), showing a monolayer of cuboidal ECs.

Scale bar in b, c, 100 µm.

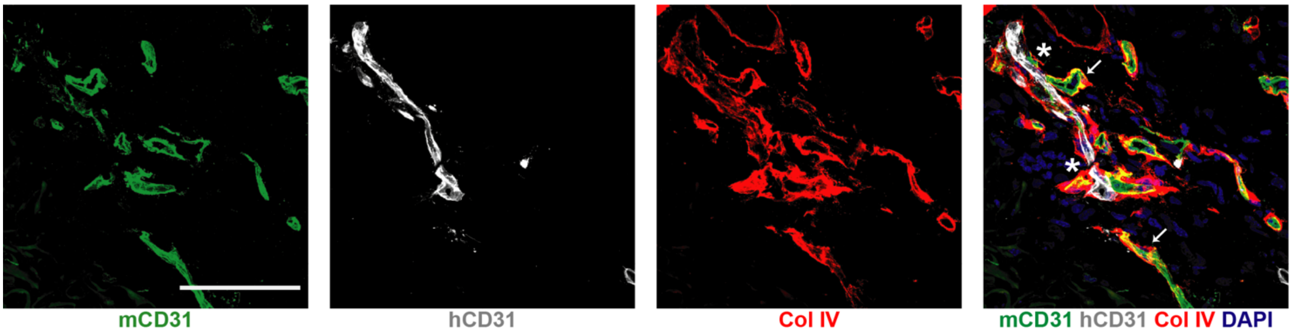

Supplementary Figure 10. Hybrid vessels formed by both SVF-derived (human) and host (mouse) ECs are surrounded by a basal membrane.

Representative image of a vascular network composed of both host ECs (mCD31$^+$, arrows) and SVF-derived ECs (hCD31$^+$, asterisks), which form hybrid vessels covered by a layer of Collagen IV$^+$ basal membrane. Scale bar, 100 µm.

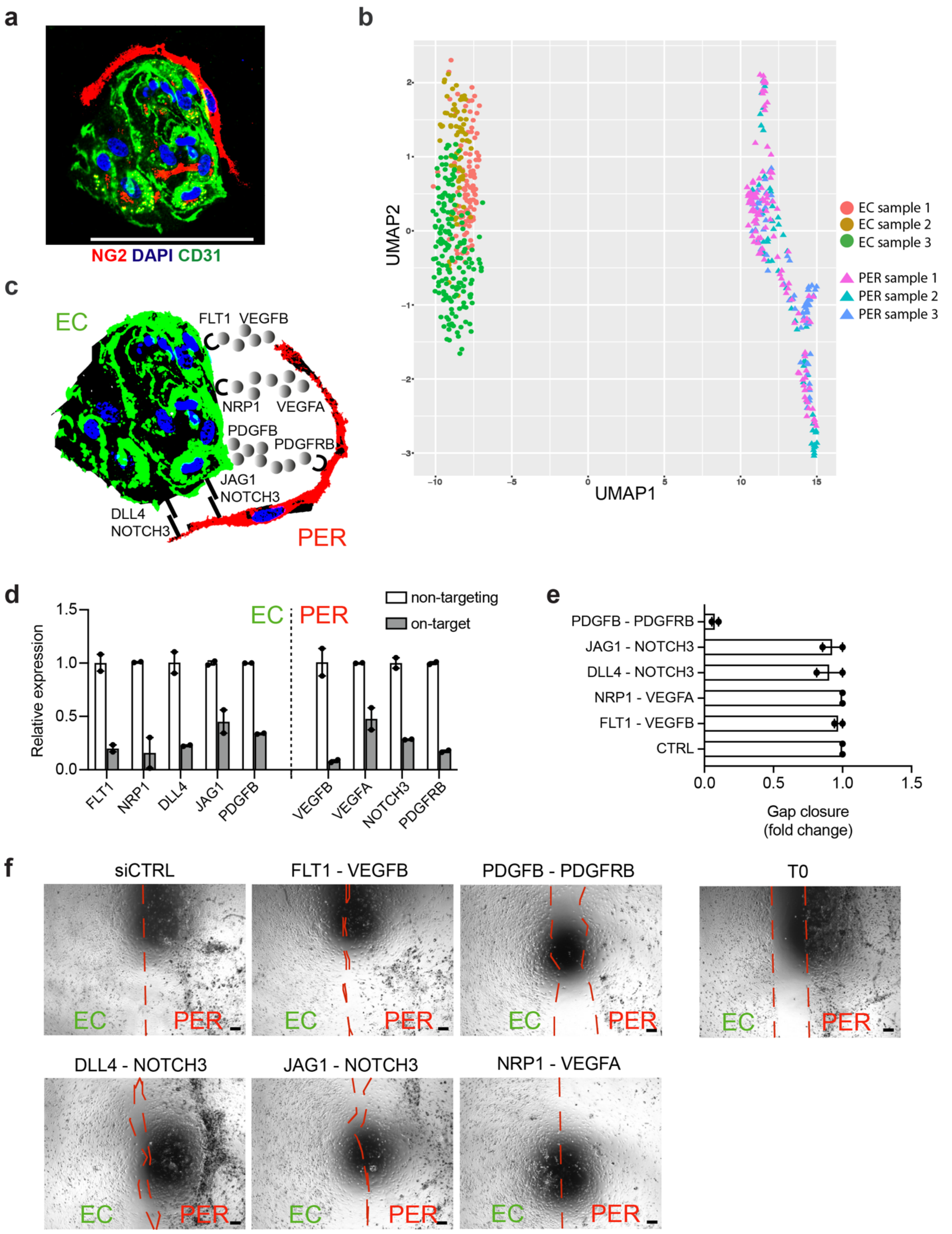

Supplementary Figure 11. PDGFB-PDGFRB is the major ligand-receptor pair mediating the interaction between ECs and pericytes from mouse SVF.

a) High magnification of SVF-derived ECs, labeled by anti-CD31 antibodies, and SVF-derived pericytes, labeled by anti-NG2 antibodies, establishing close cell-cell interactions.

b) Uniform manifold approximation and projection (UMAP) plot showing the representation of ECs (circles) and pericytes (triangles) in a 2-dimensional space, highlighting the remarkable transcriptional difference between these two cell types. Individual samples are depicted in different colours (adapted from Hildreth et al. [18]).
c) Schematic representation of green ECs and red pericytes, reproduced as binary images from real cells shown in panel a, with indication of the major ligand-receptor pairs identified by interactome analysis.
d) Real-time PCR quantification of the expression levels of the indicated genes upon delivery of either the corresponding targeting siRNAs or control, non-targeting siRNAs, in ECs and pericytes, respectively.
e) Quantification of gap closure, expressed as the percentage of the original gap area (immediately after insert removal) covered by cells at 16 hours. Data are shown as mean ± S.E.M. n = 2 per group.
f) Representative brightfield images of ECs and pericytes seeded on the two halves of a well, transfected with siRNAs specific for the ligand-receptor pair indicated on top of each image, and let to migrate to close the wound for 16 hours.

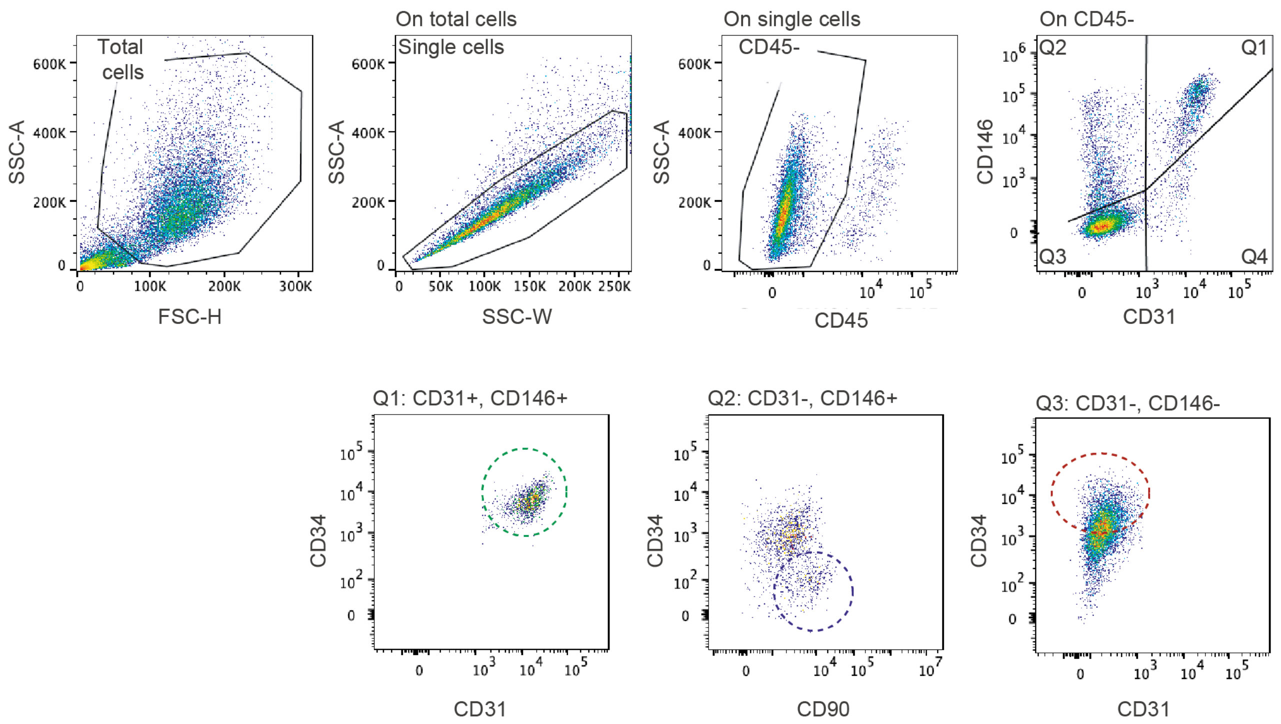

Supplementary Figure 12. Gating strategy for human SVF characterization by flow cytometry

Representative dot plots depicting the gating strategy based on physical parameters to exclude cellular debris and doublets. CD45^{-} cells were plotted according to CD31 and CD146 expression to phenotypically characterize EC (CD31^{+}CD146^{+}), pericytes (CD45^{-}CD31^{-}CD146^{+}CD34^{-}CD90^{+}) and FAPs (CD45^{-}CD31^{-}CD146^{-}CD34^{+}).

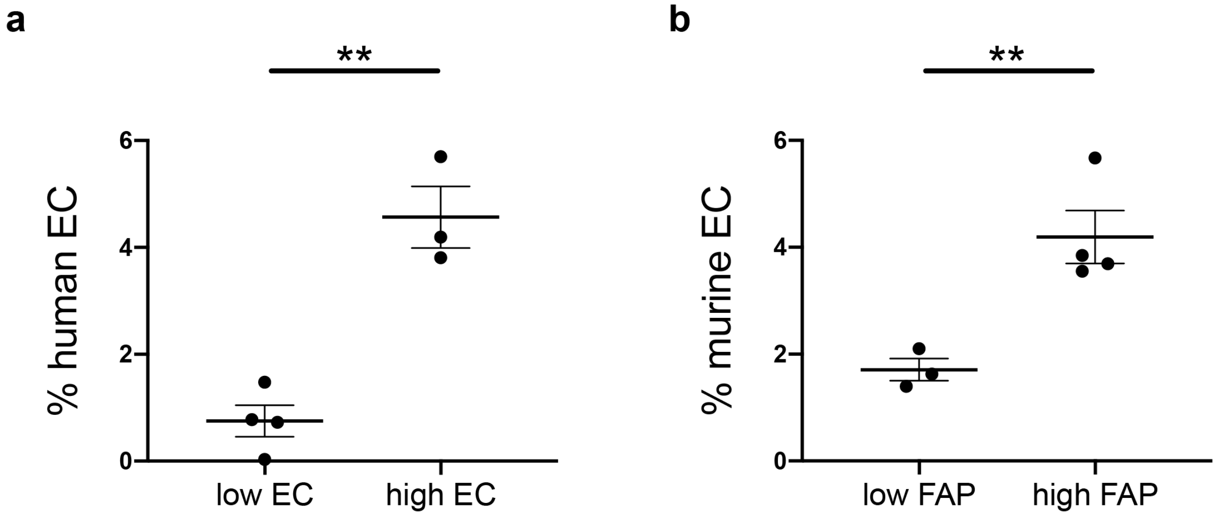

Supplementary Figure 13. SVF-derived ECs and FAPs form new vessels through direct incorporation and paracrine activity, respectively.

a) Quantification of the wound area covered by human ECs, recognized by human-specific anti-CD31 antibodies, upon implantation of INTEGRA seeded with human SVF containing either high or low number of ECs.

b) Quantification of the wound area covered by mouse ECs, recognized by mouse-specific anti-CD31 antibodies, upon implantation of INTEGRA seeded with human SVF containing either high or low number of FAPs.

Data are shown as mean ± S.E.M. n ≥ 3 per group. Statistical significance was determined using unpaired Student's t-test. **P < 0.01.

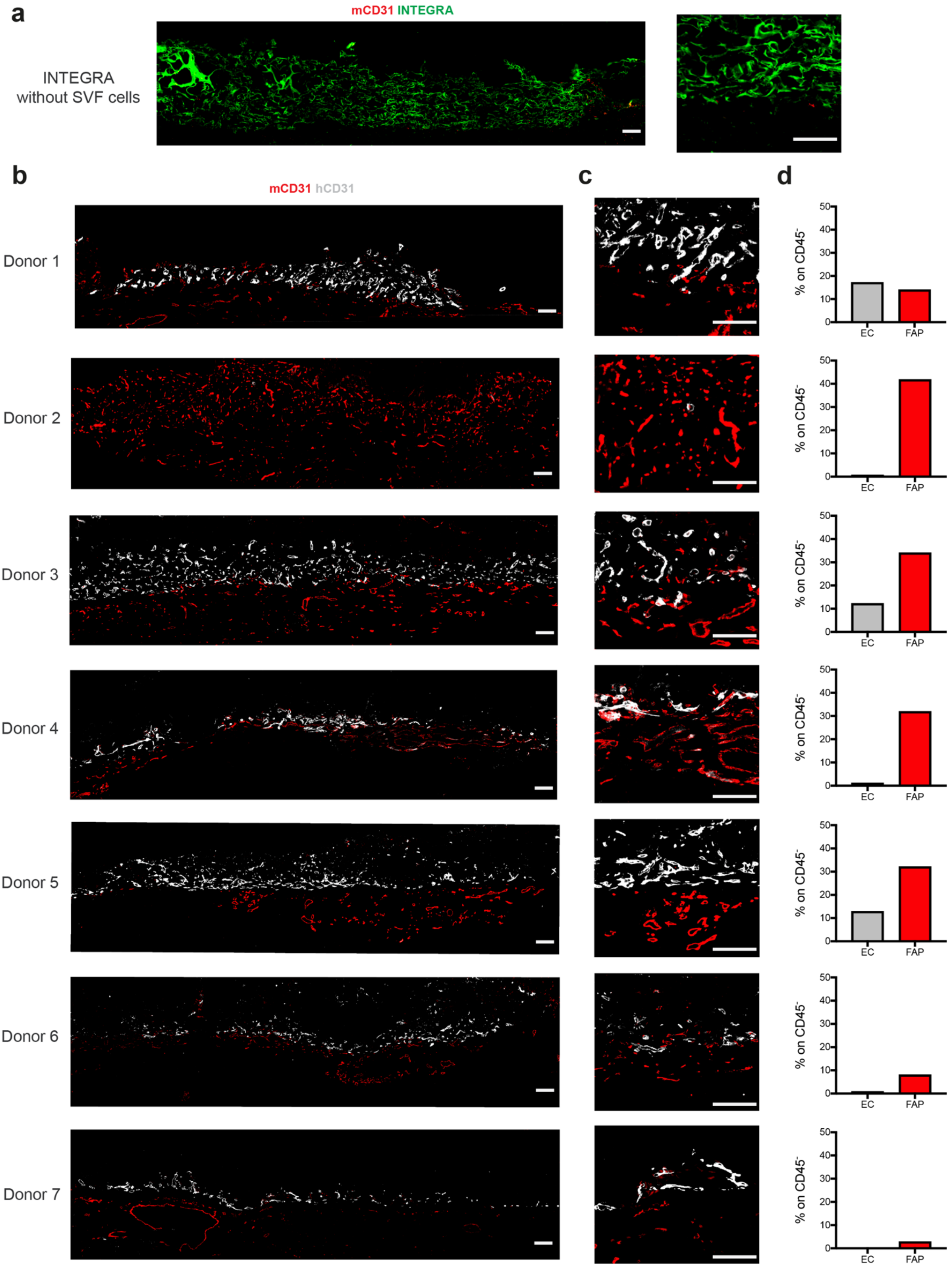

Supplementary Figure 14. Wound revascularization exerted by SVF from seven donors.

a) Low magnification image of an ischemic wound bed covered by INTEGRA without application of SVF.

b) Low magnification images of ischemic wound beds covered by INTEGRA seeded with the SVF isolated from seven human donors.
c) High magnification images showing human (white) and mouse (red) vessels colonizing the INTEGRA scaffold.
d) Quantification of the relative abundance of ECs and FAPs in the expanded SVF of each donor prior to its seeding on INTEGRA and *in vivo* implantation.

Scale bar in a, b, c, 100 µm.

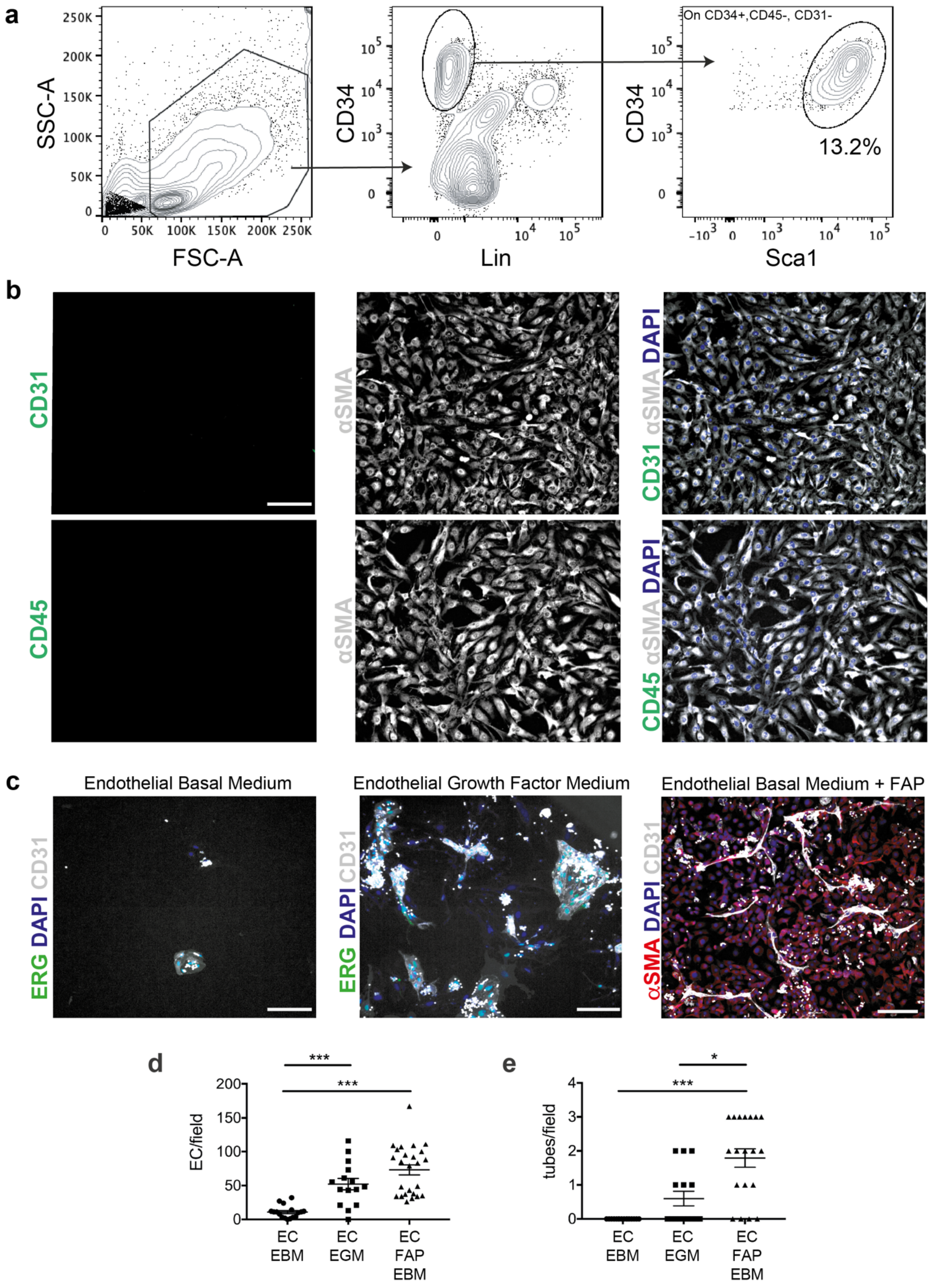

Supplementary Figure 15. Purified mouse FAPs provide trophic support to ECs.

a) Gating strategy used for the identification and sorting of mouse FAPs. Lin, lineage (CD31 and CD45).

b) Sorted FAPs were cultured for 24 hours and stained for α-SMA. The absence of $CD31^+$ and $CD45^+$ cells confirmed FAP purity and excluded contamination by either ECs or hematopoietic cells. Scale bar, 100 µm.
c) SVF-derived ECs were cultured in basal medium (EBM), growth factor-enriched medium (EGM) or basal medium in combination with SVF-derived FAPs. ECs are labeled by ERG and CD31, while FAPs are stained for α-SMA.
d) Quantification of $CD31^+$ ECs after 5 days of *ex vivo* expansion using the indicated culture media.
e) Quantification of vascular tubes formed by ECs cultured in the indicated media

Data in figure d and e are shown as mean ± S.E.M. n ≥ 3 per group. Statistical significance was determined using one-way ANOVA followed by Tukey's multiple comparison test. *$P < 0.05$, **$P < 0.01$, ***$P < 0.001$.

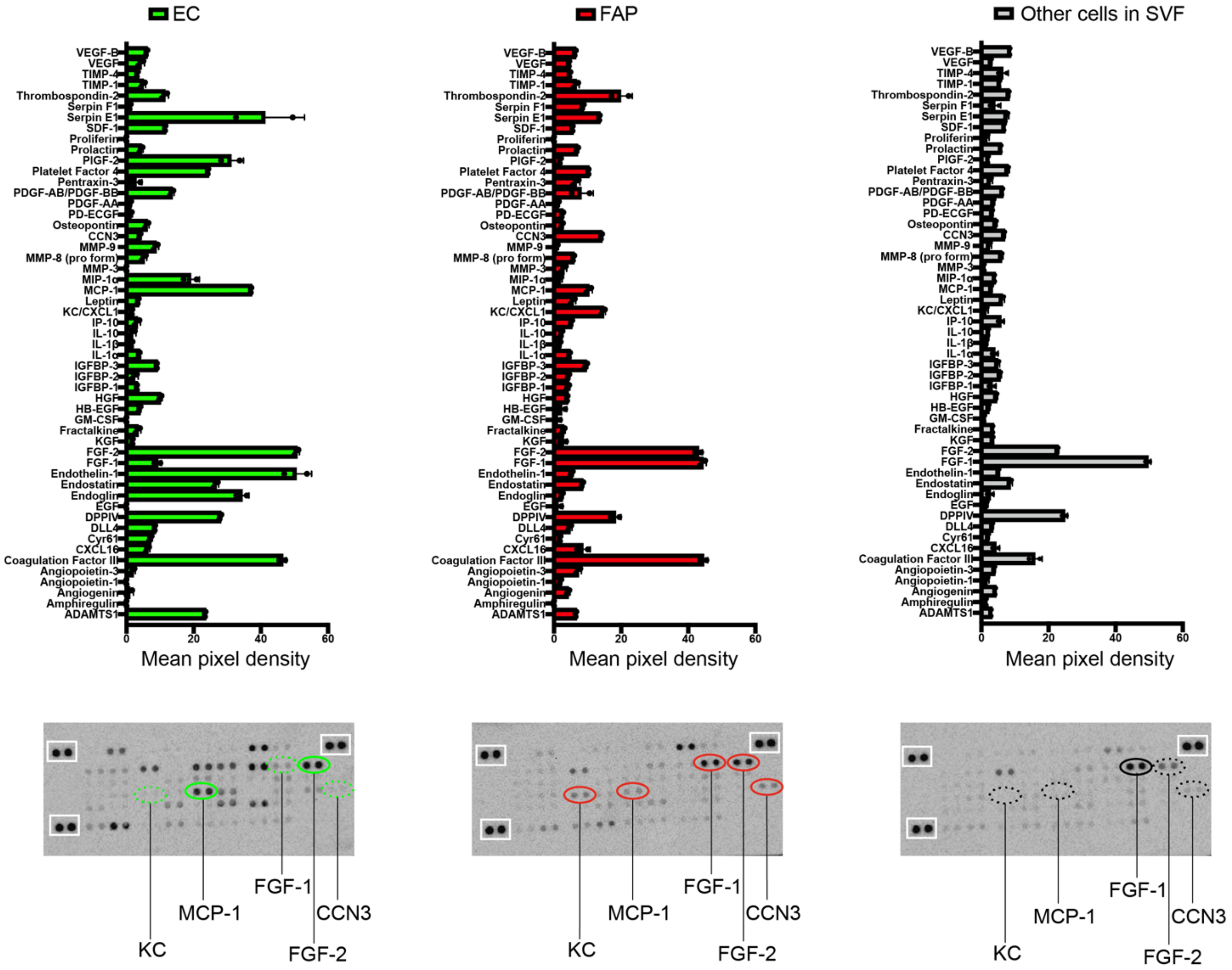

Supplementary Figure 16. Multiple pro-angiogenic factors are secreted by FAPs.
Quantification and representative images of sandwich immunoassay for the analysis of 53 angiogenesis-related proteins secreted by mouse ECs (green), FAPs (red) and the remaining cell types in the SVF (gray). Multiple pro-angiogenic factors are produced most abundantly by FAPs compared to the other cell populations. Red regular circles indicate the most abundant pro-angiogenic factors produced by FAPs. The same factors are expressed at either comparable (regular green and black circles) or lower (dashed green and black circles) levels by both ECs and other SVF cells. White boxes indicate loading controls. Data are shown as mean ± S.E.M. n = 2 per group.

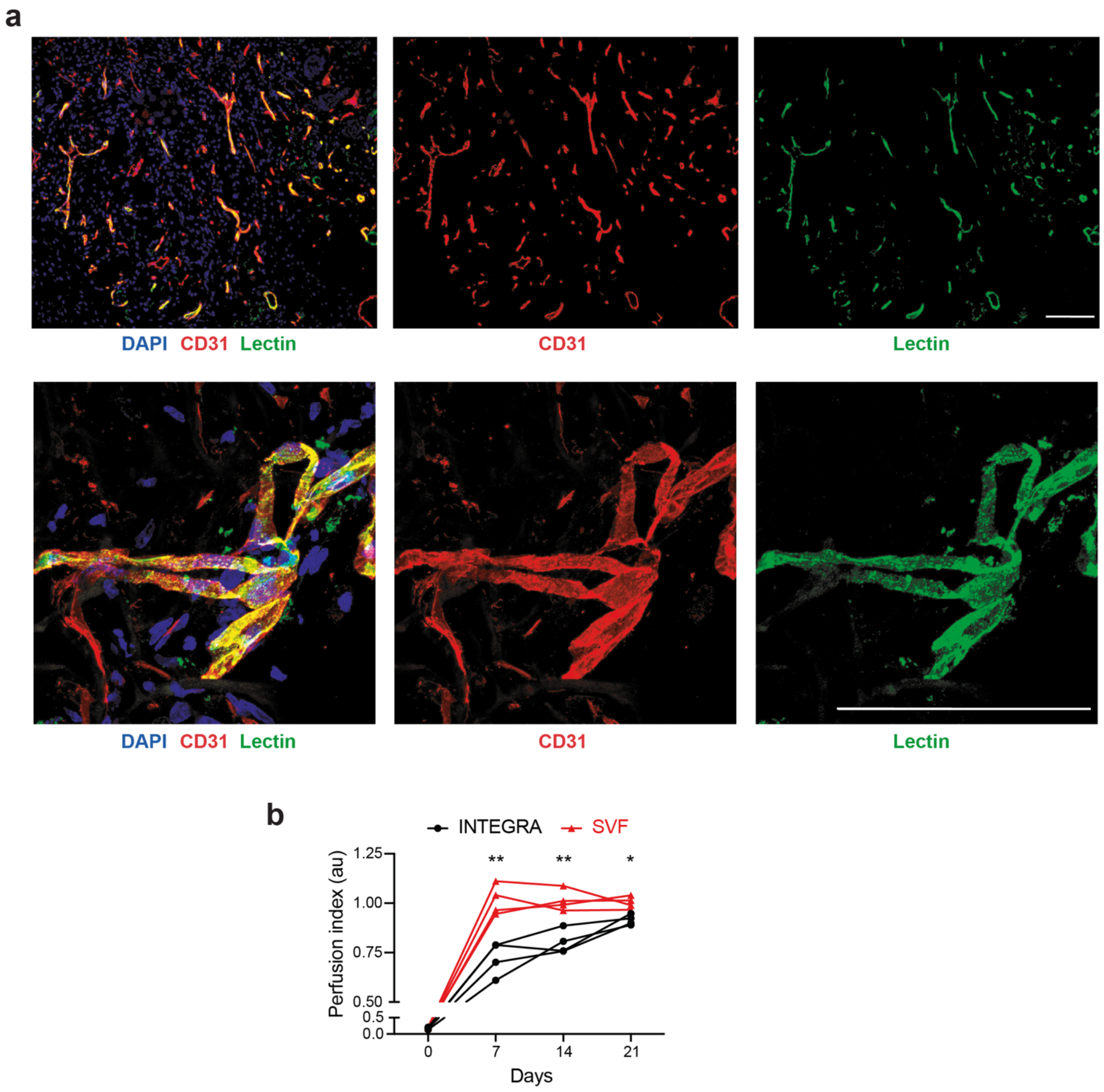

Supplementary Figure 17. Lectin labels perfused vessels.

a) Representative images of mouse subcutaneous tissue showing that all vessels perfused by lectin are $CD31^+$ at two different magnifications. Scale bar, 100 µm.

b) Quantification of wound perfusion by laser doppler analysis upon application of INTEGRA scaffold, either alone or in combination with SVF cells, at the indicated time points. Data are shown as individual values; n = 4 per group. Statistical significance was determined using two-way ANOVA for repeated measurements. *P < 0.05, **P < 0.01.

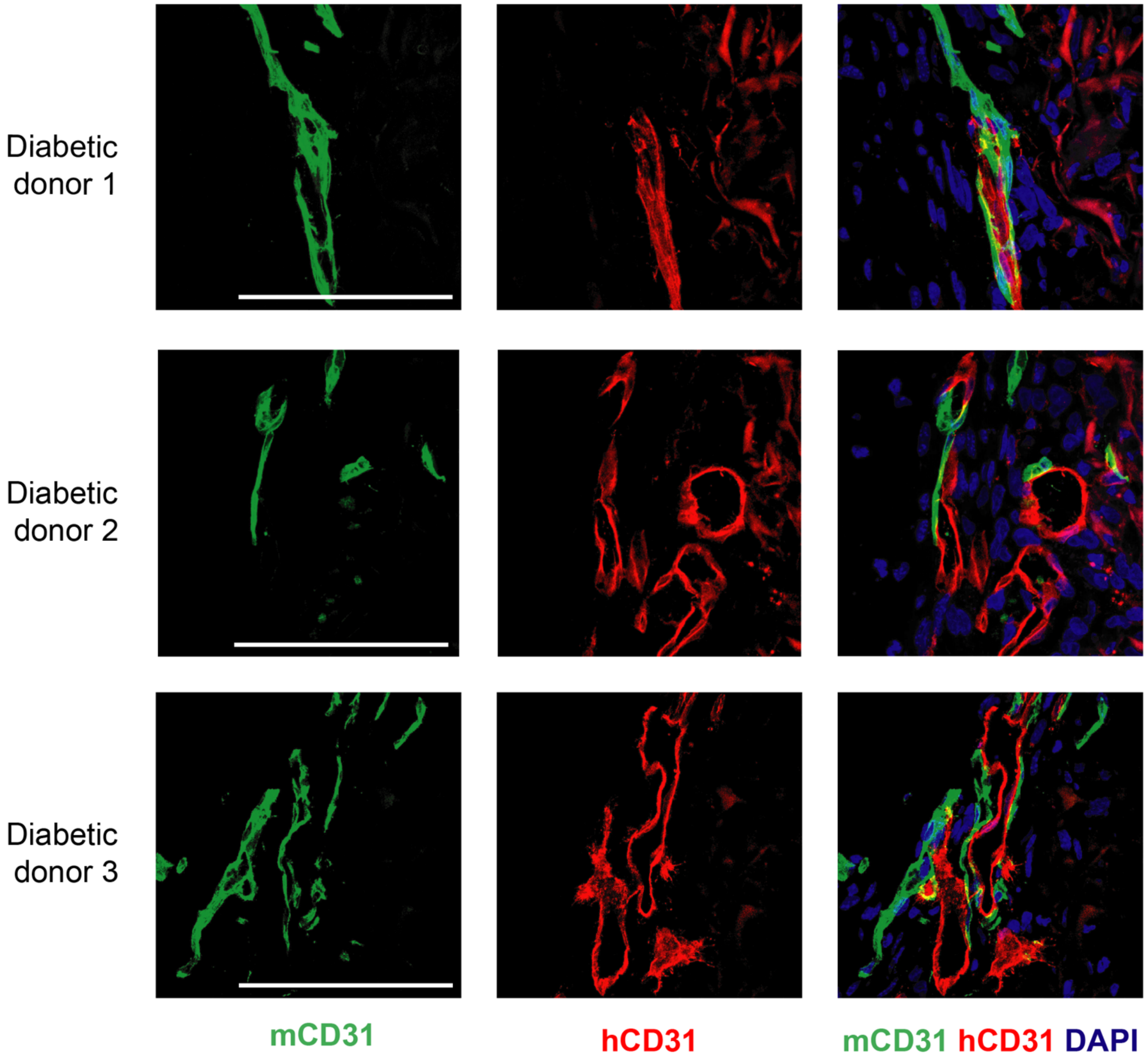

Supplementary Figure 18. The SVF derived from three diabetic donors forms hybrid vessels with host (mouse) ECs.

Representative images of three vascular networks composed of both host ECs (mCD31$^+$, green) and diabetic SVF-derived ECs (hCD31$^+$, red), forming hybrid vessels similar to what observed with SVF derived from healthy donors. Scale bar, 100 µm.

Legend to Supplementary Movies

Supplementary Movie 1. Complex vascular network formed by the SVF. The movie shows a representative 3D reconstruction of an INTEGRA sample colonized by SVF cells, in which ECs are in green and SVF remaining cells are in red, at 7 days after implantation. From the original image, isosurfaces were derived to show the composition and extension of the newly formed vascular network.

Supplementary Movie 2. SVF-derived vessels are composed by EC and perivascular cells. The movie shows a high magnification of a representative 3D reconstruction of an INTEGRA sample colonized by SVF cells, in which ECs are in green and SVF remaining cells are in red, at 7 days after implantation. From the original image, isosurfaces were derived to show red perivascular cells wrapping around SVF-derived green ECs.

Supplementary Movie 3. Hybrid vessels formed by both mouse and human ECs. The movie shows a representative 3D reconstruction of human and mouse ECs, labelled in white and red, respectively. From the original image, isosurfaces were derived to highlight the formation of hybrid vessels composed of both human and mouse ECs.

Supplementary Movie 4. A human EC generating filopodia to contact a mouse EC. The movie shows a representative 3D reconstruction of human and mouse ECs, labelled in white and red, respectively. From the original image, isosurfaces were derived to highlight the high number of filopodia emitted by a human EC sprouting to reach a mouse EC.

Supplementary Movie 5. SVF-derived vessels are perfused. The movie shows a representative 3D reconstruction of the vessels formed by the SVF, in which ECs are in green and SVF remaining cells are in red, upon injection of lectin (in white) in the recipient syngeneic animal. From the original image, isosurfaces were derived to highlight the presence of lectin inside vessels derived from SVF cells.

Supplementary Movie 6. Hybrid human-mouse vessels are perfused. The movie shows a representative 3D reconstruction of the vessels formed by human SVF, in which human ECs are in red and mouse ECs are in green, upon injection of lectin (in white) in the recipient animal. From the original image, isosurfaces were derived to highlight the presence of lectin inside vessels composed of cells of both human and mouse origin.

Supplementary Tables

Supplementary Table 1

Information on key clinical features of the donors considered in this study, together with the relative abundance of the three main SVF sub-populations (ECs, FAPs and pericytes).

	Donor	Sex	Age	BMI class	Sampling site	EC (%)	FAP (%)	PER (%)
	Donor 1	F	27	Normal weight	Thigh	17.2	14.0	14.1
	Donor 2	F	56	Obese	Abdomen	0.6	41.7	0.9
	Donor 3	F	40	Normal weight	Thigh	12.2	34.0	2.8
	Donor 4	F	61	Normal weight	Abdomen	1.0	31.9	12.0
	Donor 5	F	61	Normal weight	Hip	12.8	32.0	15.4
	Donor 6	F	44	Normal weight	Hip	0.8	8.0	28.9
	Donor 7	F	74	Normal weight	Hip	0.3	2.8	33.1
	Diabetic Donor 1	F	85	Normal weight	Abdomen	2.5	12.6	21.2
	Diabetic Donor 2	M	76	Obese	Abdomen	1.9	8.8	11.1
	Diabetic Donor 3	F	54	Obese	Abdomen	5.3	19.8	15.5

Supplementary Table 2

siRNA smart pool	Catalog number	Silenced in
siGENOME Mouse Flt1 siRNA	M-040636-01-0005	Endothelial cells
siGENOME Mouse Nrp1 siRNA	M-040787-00-0005	Endothelial cells
siGENOME Mouse Dll4 siRNA	M-045947-02-0005	Endothelial cells
siGENOME Mouse Jag1 siRNA	M-041922-01-0005	Endothelial cells
siGENOME Mouse Pdgfb siRNA	M-050604-01-0005	Endothelial cells
siGENOME Mouse Vegfb siRNA	M-047407-01-0005	Pericytes
siGENOME Mouse Vegfa siRNA	M-040812-01-0005	Pericytes
siGENOME Mouse Notch3 siRNA	M-047867-01-0005	Pericytes
siGENOME Mouse Pdgfrb siRNA	M-048218-00-0005	Pericytes

Supplementary Table 3

Antibody	Company	Catalog number
Goat monoclonal antibody anti-mouse CD31/PECAM-1	R&D/Bio-Techne	#AF3628
Mouse monoclonal antibody anti-human CD31/PECAM-1	Dako	#M0823
Rabbit polyclonal antibody anti-laminin	Sigma-Aldrich	#L9393
Monoclonal antibody Anti-Actin, alpha-Smooth Muscle - Cy3	Sigma-Aldrich	#C6198
Mouse monoclonal antibody anti-Actin, alpha-Smooth Muscle	Dako	#M0823
Goat polyclonal biotinylated antibody anti-mouse PDGF-R beta	R&D/Bio-Techne	#BAF1042
Rabbit polyclonal antibody anti-NG2 Chondroitin Sulfate Proteoglycan	Merck	#AB5320
Rabbit monoclonal antibody anti-LYVE1	Abcam	#ab218535
Goat polyclonal IgG antibody anti-mouse EphB4	R&D/Bio-Techne	#AF446
Lectin-biotin conjugate from Lycopersicon esculentum	VectorLab / DBA	#B-1175-1

Goat polyclonal antibody anti-GFP	Abcam	#ab5450
Rabbit monoclonal antibody anti-Ki67	Cell signaling	#9129

Supplementary Table 4

**Antibody**	**Company**	**Catalog number**
Alexa Fluor 647 Donkey anti-mouse IgG (H+L)	Invitrogen	#A-31571
Alexa Fluor 594 Donkey anti-mouse IgG (H+L)	Invitrogen	#A-32740
Alexa Fluor 647 Donkey anti-goat IgG (H+L)	Invitrogen	#A-21447
Alexa Fluor 594 Donkey anti-goat IgG (H+L)	Invitrogen	#A-11058
Alexa Fluor 488 Donkey anti-goat IgG (H+L)	Invitrogen	#A-11055
Alexa Fluor 647 Donkey anti-rabbit IgG (H+L)	Invitrogen	#A-31573
Alexa Fluor 594 Donkey anti-rabbit IgG (H+L)	Invitrogen	#A-21207
Alexa Fluor 488 Donkey anti-rabbit IgG (H+L)	Invitrogen	#A-21206
Streptavidin APC Conjugate	eBioscience™	#17-4317-82
Streptavidin, Alexa Fluor® 546 conjugate	ThermoFischer	#S11225

Supplementary Table 5

Gene	Forward	Reverse	Assessed in
Flt1	TTCATCAGTGTGAAACATCG	CGAGCCATCTTTTAACCATAC	Endothelial cells
Nrp1	AACAGGTGGAGGCATGGGGACT	ATCGTGGATGCCCTTCCGAGCT	Endothelial cells
Dll4	GCGGAGGAGGAGGAAATGGCA	TGCCCATCTGAAGCCAGGCAGA	Endothelial cells
Jag1	AAACACGGAGCCAACACGGTCC	TGGCTGTTTGGCAAAGCGGACT	Endothelial cells
Pdgfb	TGCAACGAGAAAGCCGGAGCAG	ATCTACCCACTCGCTCGCCACT	Endothelial cells
Vegfb	GATCCTCATGATCCAGTACC	TTTGGTCTGCATTCACATTG	Pericytes
Vegfa	GGAGTACCCCGACGAGATAG	CTCACAGTGATTTTCTGGCTTT	Pericytes
Notch3	AAGAGACGCCGCTGTTCTTGGC	TCCCGGTTGGCGAGATGATCCA	Pericytes
Pdgfrb	TGGAGCCCGGATTCCTGCAGAA	AGAGCAATCCAGCTGAGGGGCA	Pericytes
Gapdh	ACAACTTTGGCATTGTGGAA	GATGCAGGGATGATGTTCTG	Both
