# Supplementary material for: Ischemic wound revascularization by the stromal vascular fraction relies on host-donor hybrid vessels
Source: NPJ Regen Med. 2023 Feb 11;8:8. doi: 10.1038/s41536-023-00283-6 (PMC9922297; doi:10.1038/s41536-023-00283-6)
Supplement: Supplementary file 9 — Reporting Summary [file 41536_2023_283_MOESM9_ESM.pdf]

nature portfolio

Corresponding author(s): Serena Zacchigna
NPJREGENMED-01138

Last updated by author(s): Nov 21, 2022

Reporting Summary

Nature Portfolio wishes to improve the reproducibility of the work that we publish. This form provides structure for consistency and transparency in reporting. For further information on Nature Portfolio policies, see our Editorial Policies and the Editorial Policy Checklist.

Statistics

For all statistical analyses, confirm that the following items are present in the figure legend, table legend, main text, or Methods section.

n/a	Confirmed	
☐	☒	The exact sample size (*n*) for each experimental group/condition, given as a discrete number and unit of measurement
☒	☐	A statement on whether measurements were taken from distinct samples or whether the same sample was measured repeatedly
☐	☒	The statistical test(s) used AND whether they are one- or two-sided<br>*Only common tests should be described solely by name; describe more complex techniques in the Methods section.*
☒	☐	A description of all covariates tested
☐	☒	A description of any assumptions or corrections, such as tests of normality and adjustment for multiple comparisons
☐	☒	A full description of the statistical parameters including central tendency (e.g. means) or other basic estimates (e.g. regression coefficient) AND variation (e.g. standard deviation) or associated estimates of uncertainty (e.g. confidence intervals)
☐	☒	For null hypothesis testing, the test statistic (e.g. *F*, *t*, *r*) with confidence intervals, effect sizes, degrees of freedom and *P* value noted<br>*Give P values as exact values whenever suitable.*
☒	☐	For Bayesian analysis, information on the choice of priors and Markov chain Monte Carlo settings
☒	☐	For hierarchical and complex designs, identification of the appropriate level for tests and full reporting of outcomes
☒	☐	Estimates of effect sizes (e.g. Cohen's *d*, Pearson's *r*), indicating how they were calculated

Our web collection on statistics for biologists contains articles on many of the points above.

Software and code

Policy information about availability of computer code

Data collection	Microsoft Excel Office 2019
Data analysis	Graphpad Prism 8, DaMiRseq-an R/Bioconductor package, CellPhoneDB (v.2.1.7), FlowJo (v.10), ImageJ/Fiji (v.2.1.0/1.53c)

For manuscripts utilizing custom algorithms or software that are central to the research but not yet described in published literature, software must be made available to editors and reviewers. We strongly encourage code deposition in a community repository (e.g. GitHub). See the Nature Portfolio guidelines for submitting code & software for further information.

Data

Policy information about availability of data

All manuscripts must include a data availability statement. This statement should provide the following information, where applicable:

- Accession codes, unique identifiers, or web links for publicly available datasets
- A description of any restrictions on data availability
- For clinical datasets or third party data, please ensure that the statement adheres to our policy

Source data are provided with this paper.

Human research participants

Policy information about studies involving human research participants and Sex and Gender in Research.

Reporting on sex and gender	The sex (biological attribute) is indicated in this paper. The information has been collected to describe the donor of biological material.
Population characteristics	*Describe the covariate-relevant population characteristics of the human research participants (e.g. age, genotypic information, past and current diagnosis and treatment categories). If you filled out the behavioural & social sciences study design questions and have nothing to add here, write "See above."*
Recruitment	*Describe how participants were recruited. Outline any potential self-selection bias or other biases that may be present and how these are likely to impact results.*
Ethics oversight	The collection and manipulation of human samples was performed upon approval by the competent Ethical Committee (authorization n. 15569/P/GEN/ARCS) and obtainment of informed consent by the patient.

Note that full information on the approval of the study protocol must also be provided in the manuscript.

Field-specific reporting

Please select the one below that is the best fit for your research. If you are not sure, read the appropriate sections before making your selection.

☒ Life sciences ☐ Behavioural & social sciences ☐ Ecological, evolutionary & environmental sciences

For a reference copy of the document with all sections, see nature.com/documents/nr-reporting-summary-flat.pdf

Life sciences study design

All studies must disclose on these points even when the disclosure is negative.

Sample size	Sample size for animal experiments was determined using www.gpower.hhu.de to detect an effect with power = 0.8
Data exclusions	No data were excluded from the analyses of this work
Replication	All experiments in the main figures have been successfully repeated at least three times.
Randomization	Animals involved in the same experiments were all syngenic and of the same age, so they have been randomly allocated in the different experimental groups.
Blinding	All the processes of this work, from group allocation to data collection and analysis, have been performed in blind.

Reporting for specific materials, systems and methods

We require information from authors about some types of materials, experimental systems and methods used in many studies. Here, indicate whether each material, system or method listed is relevant to your study. If you are not sure if a list item applies to your research, read the appropriate section before selecting a response.

Materials & experimental systems

n/a	Involved in the study	
☐	☒	Antibodies
☒	☐	Eukaryotic cell lines
☒	☐	Palaeontology and archaeology
☐	☒	Animals and other organisms
☒	☐	Clinical data
☒	☐	Dual use research of concern

Methods

n/a	Involved in the study	
☒	☐	ChIP-seq
☐	☒	Flow cytometry
☒	☐	MRI-based neuroimaging

Antibodies

Antibodies used	1) Mouse CD31/PECAM-1 (R&D/Bio-Techne, #AF3628) at 1:100<br>2) Human CD31/PECAM-1 (Dako, #M0823) at 1:100

3) Laminin (Sigma-Aldrich, #L9393)
4) Actin, alpha-Smooth Muscle - Cy3 (Sigma-Aldrich, #C6198) at 1:500
5) Actin, alpha-Smooth Muscle (Dako, #M0823) at 1:100
6) PDGF-R beta (R&D/Bio-Techne, #BAF1042)
7) NG2 Chondroitin Sulfate Proteoglycan (Merck, #AB5320) at 1:100
8) LYVE1 (Abcam, #ab218535) at 1:100
9) EphB4 (R&D/Bio-Techne, #AF446) at 1:100
10) Lectin from Lycopersicon esculentum (VectorLab / DBA, #B-1175-1) IV injected 1:1, final volume 100 µL
11) GFP (Abcam, #ab5450) at 1:100
12) Ki67 (Cell signaling, #9129) at 1:100
13) CD34 PE (clone 4H11, Invitrogen, 12-0349-42) at 1:500
14) CD146 Ax647 (clone P1H12, Biolegend, 361013) at 1:500
15) CD45 BV421 (clone H130, BD Horizon, 563880) at 1:500
16) CD31 FITC (clone WM59, Invitrogen 11-039-42) at 1:500
17) CD90 BV786 (clone 5E10, BD OptiBuild, 740986) at 1:500.

Validation

All commercial antibodies were validated by the manufacturer for the species and application used in this study.
1) Mouse CD31/PECAM-1 (R&D/Bio-Techne, #AF3628) https://www.biotechne.com/p/antibodies/mouse-rat-cd31-pecam-1-antibody_af3628
2) human CD31/PECAM-1 (Dako, #M0823)
https://www.agilent.com/en/product/immunohistochemistry/antibodies-controls/primary-antibodies/cd31-endothelial-cell-(concentrate)-76539
3) Laminin (Sigma-Aldrich, #L9393)
https://www.sigmaaldrich.com/IT/it/product/sigma/l9393?gclid=EAIaIQobChMIlu6Qgsi8-wIVSf7VCh15BQyREAAYASAAEgLGfvD_BwE&gclsrc=aw.ds
4) Actin, alpha-Smooth Muscle - Cy3 (Sigma-Aldrich, #C6198) https://www.sigmaaldrich.com/IT/it/product/sigma/c6198
5) Actin, alpha-Smooth Muscle (Dako, #M0823)
https://www.agilent.com/en/product/immunohistochemistry/antibodies-controls/primary-antibodies/actin-(smooth-muscle)-(concentrate)-76542
6) PDGF-R beta (R&D/Bio-Techne, #BAF1042) https://www.rndsystems.com/products/mouse-pdgf-rbeta-biotinylated-antibody_baf1042
7) NG2 Chondroitin Sulfate Proteoglycan (Merck, #AB5320) https://www.merckmillipore.com/IT/it/product/Anti-NG2-Chondroitin-Sulfate-Proteoglycan-Antibody,MM_NF-AB5320
8) LYVE1 (Abcam, #ab218535)
https://www.abcam.com/lyve1-antibody-epr21771-ab218535.html
9) EphB4 (R&D/Bio-Techne, #AF446)
https://www.rndsystems.com/products/mouse-ephb4-antibody_af446
10) Lectin from Lycopersicon esculentum (VectorLab / DBA, #B-1175-1) https://vectorlabs.com/products/glycobiology/biotinylated-lycopersicon-tomato-lectin
11) GFP (Abcam, #ab5450)
https://www.abcam.com/gfp-antibody-ab5450.html
12) Ki67 (Cell signaling, #9129)
https://www.cellsignal.com/products/primary-antibodies/ki-67-d3b5-rabbit-mab/9129
13) CD34 PE (clone 4H11, Invitrogen, 12-0349-42) https://www.thermofisher.com/antibody/product/CD34-Antibody-clone-4H11-Monoclonal/12-0349-42
14) CD146 Ax647 (clone P1H12, Biolegend, 361013)
https://www.biolegend.com/it-it/products/apcfire750-anti-human-cd146-antibody-16597
15) CD45 BV421 (clone H130, BD Horizon, 563880)
https://www.bdbiosciences.com/en-au/products/reagents/flow-cytometry-reagents/research-reagents/single-color-antibodies-ruo/bv421-mouse-anti-human-cd45.563880
16) CD31 FITC (clone WM59, Invitrogen 11-039-42) https://www.thermofisher.com/antibody/product/CD31-PECAM-1-Antibody-clone-WM-59-WM59-Monoclonal/11-0319-42
17) CD90 BV786 (clone 5E10, BD OptiBuild, 740986)
https://www.bdbiosciences.com/en-us/products/reagents/flow-cytometry-reagents/research-reagents/single-color-antibodies-ruo/bv786-mouse-anti-human-cd90.740986

Animals and other research organisms

Policy information about studies involving animals; ARRIVE guidelines recommended for reporting animal research, and Sex and Gender in Research

Laboratory animals

BALB/C (8 weeks old, male), C57BL/6 (48 weeks old, male) and NSG (48 weeks old, male), Apln-CreER/mTmG (8 weeks old, background C57BL/6, male), Cdh5-CreER/mTmG (8 weeks old, background C57BL/6, male), mTmG (8 weeks old, background C57BL/6, male).

Wild animals

The study did not involve wild animals.

Reporting on sex

Sex differences were not considered in this study.

Field-collected samples

The study did not involve field-collected samples.

Ethics oversight

Animals were housed in compliance with institutional guidelines from the Directive 2010/63/EU of the European Parliament on

Ethics oversight	animal experimentation in compliance with European guidelines and International Laws and Policies (EC Council Directive 86/609, OJL 34, 12 December 1987). All experimental procedures were approved by the ICGEB Animal Welfare Board, as required by the EU Directive 2010/63/EU, and by the Italian Ministry of Health (authorization n. 213/2022-PR).

Note that full information on the approval of the study protocol must also be provided in the manuscript.

Flow Cytometry

Plots

Confirm that:

☒ The axis labels state the marker and fluorochrome used (e.g. CD4-FITC).

☒ The axis scales are clearly visible. Include numbers along axes only for bottom left plot of group (a 'group' is an analysis of identical markers).

☒ All plots are contour plots with outliers or pseudocolor plots.

☒ A numerical value for number of cells or percentage (with statistics) is provided.

Methodology

Sample preparation	Mouse SVF was isolated from inguinal white adipose tissue of adult BALB/C, mTmG, Cdh5-CreER/mTmG or Apln-CreER/mTmG mice. Human SVF was isolated from subcutaneous lipoaspirate of thigh, abdomen or hip. The SVF of diabetic patients was isolated from the abdominal subcutaneous adipose tissue. The adipose tissue was rinsed with calcium- and bicarbonate-free Hank's solution with HEPES (CBFHH) and minced using fine scissors until the tissue suspension appeared homogeneous. The tissue was transferred in gentleMACS C Tubes (Miltenyi Biotec, 130-093-237, 130-096-334) containing a pre-warmed digestion solution composed of 1 mg/mL Collagenase NB4 Standard Grade (Nordmark Biochemicals, S1745401), 0.5 mg/mL DNase II (Sigma, D8764) and 100 µg/mL antibiotics (gentamicin, penicillin/streptomycin) dissolved in BFHH (Bicarbonate-Free Hanks´solution with HEPES) supplemented with 2mM CaCl2. GentleMACS™ Octo Dissociator with Heaters (Miltenyi Biotec, 130-096-427) was used to digest the tissue running the program 37C_mr_ATDK_1. The digested tissue was then diluted in Dulbecco's Modified Eagle Medium (DMEM, Sigma) supplemented with 10% foetal bovine serum (FBS) and centrifuged at 700 x g for 10 minutes. After removing the oily and liquid layers, the pellet was resuspended in DMEM with 10% FBS and filtered using a 70 µm pore size cell strainer (Falcon, 352350). The cell suspension was centrifuged at 500 x g for 8 minutes and resuspended in FACS buffer with human Fc blocking antibody (dilution 1:100, BD Pharmigen, 564219). After 10 minutes, the cells were centrifuged at 500 x g for 5 minutes and resuspended in staining-cocktail for 25 minutes on ice, protected from light. After incubation, cells were rinsed twice with FACS buffer and fixed in 4% PFA for acquisition.
Instrument	Aria cytometer (FACS ARIA II BD), Facs Celesta (BD Biosciences).
Software	Flow data were analyzed with Diva software (BD Bioscience) or FlowJo software (Tree Star, Inc.).
Cell population abundance	The purity of sorted cells was determined by post-sort analysis through immunofluorescence analysis as shown in Supplementary Fig 12B.
Gating strategy	Gating strategies is described in Figure 4I and Supplementary Fig 1F and 12A. Gates are always based on initial debris and doublets exclusion based on physical parameters. Gates are drawn on FMO controls.

☒ Tick this box to confirm that a figure exemplifying the gating strategy is provided in the Supplementary Information.
